# Chicken Production and Human Clinical *Escherichia coli* Isolates Differ in Their Carriage of Antimicrobial Resistance and Virulence Factors

**DOI:** 10.1128/aem.01167-22

**Published:** 2023-01-18

**Authors:** Reed Woyda, Adelumola Oladeinde, Zaid Abdo

**Affiliations:** a Program of Cell and Molecular Biology, Colorado State University, Fort Collins, Colorado, USA; b U.S. National Poultry Research Center, USDA-ARS, Athens, Georgia, USA; c Department of Microbiology, Immunology, and Pathology, Colorado State University, Fort Collins, Colorado, USA; Unversidad de los Andes

**Keywords:** *Escherichia coli*, antimicrobial resistance, poultry production, random forests, virulence factors

## Abstract

Contamination of food animal products by Escherichia coli is a leading cause of foodborne disease outbreaks, hospitalizations, and deaths in humans. Chicken is the most consumed meat both in the United States and across the globe according to the U.S. Department of Agriculture. Although E. coli is a ubiquitous commensal bacterium of the guts of humans and animals, its ability to acquire antimicrobial resistance (AMR) genes and virulence factors (VFs) can lead to the emergence of pathogenic strains that are resistant to critically important antibiotics. Thus, it is important to identify the genetic factors that contribute to the virulence and AMR of E. coli. In this study, we performed in-depth genomic evaluation of AMR genes and VFs of E. coli genomes available through the National Antimicrobial Resistance Monitoring System GenomeTrackr database. Our objective was to determine the genetic relatedness of chicken production isolates and human clinical isolates. To achieve this aim, we first developed a massively parallel analytical pipeline (Reads2Resistome) to accurately characterize the resistome of each E. coli genome, including the AMR genes and VFs harbored. We used random forests and hierarchical clustering to show that AMR genes and VFs are sufficient to classify isolates into different pathogenic phylogroups and host origin. We found that the presence of key type III secretion system and AMR genes differentiated human clinical isolates from chicken production isolates. These results further improve our understanding of the interconnected role AMR genes and VFs play in shaping the evolution of pathogenic E. coli strains.

**IMPORTANCE** Pathogenic Escherichia coli causes disease in both humans and food-producing animals. E. coli pathogenesis is dependent on a repertoire of virulence factors and antimicrobial resistance genes. Food-borne outbreaks are highly associated with the consumption of undercooked and contaminated food products. This association highlights the need to understand the genetic factors that make E. coli virulent and pathogenic in humans and poultry. This research shows that E. coli isolates originating from human clinical settings and chicken production harbor different antimicrobial resistance genes and virulence factors that can be used to classify them into phylogroups and host origins. In addition, to aid in the repeatability and reproducibility of the results presented in this study, we have made a public repository of the Reads2Resistome pipeline and have provided the accession numbers associated with the E. coli genomes analyzed.

## INTRODUCTION

Escherichia coli are ubiquitous commensal bacteria in the gut of both humans and food-producing animals and rarely cause disease but may acquire antimicrobial resistance (AMR) genes and virulence factors (VFs) resulting in increased pathogenicity (1). Pathogenic E. coli has consistently ranked in the top five causative agents of disease outbreaks, outbreak-associated illnesses, and hospitalizations in the United States (2–4) and is responsible for billions of dollars of annual health care associated costs in the United States (5, 6). Pathogenic E. coli infections in humans may result in acute to severe diarrhea or dysentery, urinary tract infections, and meningitis (7–9). E. coli pathogenesis is dependent on the VF repertoire, which enables the bacterium to evade host defenses, adhere to host surfaces, and successfully invade—and replicate in—host tissues. For example, VFs such as toxins, iron-acquisition systems, and fimbriae play integral roles in the pathogenicity of extraintestinal E. coli strains that cause urinary tract infections in humans (10). These uropathogenic E. coli strains are able to colonize human mucosal surfaces due to surface adhesion VFs, including the P, F, and type 1 fimbriae encoded by *pap*, *sfa*, and *fim* genes (11).

Outbreaks in human populations caused by pathogenic E. coli are attributed to the consumption of undercooked and contaminated foods, including meats and vegetables (12). E. coli infections in food-producing animals usually results in diarrhea but can include acute fatal septicemia, airsacculitis, pericarditis, and perihepatitis (13). In poultry, avian-pathogenic E. coli infections, termed avian colibacillosis, can result in economic loss due to the cost of treatment, as well as from culling of flocks (14). Colibacillosis is a leading cause of morbidity and mortality in poultry, as noted by decreases in the feed conversion ratio, egg production, and hatching rates (15–17). The severity of the disease depends on the VF repertoire of the strain, including genes encoding iron acquisition and transport systems (18).

Extraintestinal pathogenic E. coli strains that cause urinary tract infections, neonatal meningitis, and sepsis have VFs similar to those of E. coli isolates from meat and animal sources (19). Likewise, some avian-pathogenic E. coli and extraintestinal pathogenic E. coli isolates have been reported to share similar VFs and belong to the same multilocus sequence type (MLST) and phylogroup (20–23). Specifically, a microarray-based study of E. coli isolates from both human and animal sources in Denmark identified 66 to 87 genes, including both virulence factors and antimicrobial resistance genes, which were present both in human urinary tract infection isolates and in isolates obtained from poultry and pig products (24). Genes such as the iron acquisition genes *iutA* and *iroN*, as well as fimbrial *papA* encoding the Pap fimbrial major subunit and adhesion genes (e.g., *papGII* encoding Pap adhesion), were detected in both urinary tract infection and poultry-related isolates (24). *In vivo* and *in vitro* experimental studies have shown that extraintestinal pathogenic E. coli recovered from humans can cause disease in avian hosts and, similarly, avian-pathogenic E. coli isolates recovered from avian host can cause disease in mammalian models (25–29).

Infections with pathogenic E. coli are commonly treated with antibiotics; however, increasing levels of AMR impose difficulties in selecting effective treatment options (30). The spread and increased prevalence of AMR has been linked to both the overuse and the misuse of antibiotics in human clinical settings, as well as in food animal production (31). Consumer opinion has led to a reduction in antimicrobial use in food animal production to mitigate the spread of AMR. However, recent studies have indicated that AMR can still persist in food animal production even after the removal or stoppage of antibiotics (32, 33). The comobilization and coacquisition of AMR genes and VFs through horizontal gene transfer can result in highly pathogenic E. coli strains. Recent studies have demonstrated a correlation and close genetic linkage of AMR genes and VFs in pathogenic bacterial strains (34, 35). Pan et al. (35) performed a comprehensive analysis of over 9,000 bacterial genomes from multiple species and hosts and observed the coexistence of AMR genes and VFs from human-associated pathogens. Therefore, the aim of this study was to evaluate and compare the current distribution of AMR genes and VFs present in E. coli isolates from chicken production and human clinical settings in the United States.

To do so, we determined the AMR genes and VFs present in nearly 800 E. coli genomes collected from chicken production and the human clinical settings. Data for all isolates were obtained from the National Antimicrobial Resistance Monitoring System (NARMS). We used the most recent World Health Organization (WHO) classification of antimicrobials important to human health to determine the extent to which resistance to these antimicrobials are prevalent in chicken production and human clinical settings. We hypothesized that resistance to antimicrobials of critical and high importance to human health would be present in both human- and chicken-derived isolates. In addition, we hypothesized that E. coli isolates from human clinical settings and chicken production will differ in their carriage of AMR genes and VFs. We found that E. coli isolates obtained from chicken production and human clinical settings harbored AMR genes predicted to confer resistance to many antimicrobials classified as highly and critically important to human health by the WHO. Furthermore, we were able to classify all E. coli isolates into their respective phylogroups and host origins using random forest classification and hierarchical clustering of the AMR genes and VFs found in their genomes.

## RESULTS

### *E. coli* isolate selection and genome assembly.

To evaluate and compare the current distribution of AMR genes and VFs present in E. coli isolates from chicken production and the human clinical settings in the United States, we took advantage of the U.S. National Antimicrobial Resistance Monitoring System (NARMS) GenomeTrackr database (available through the NCBI [https://www.ncbi.nlm.nih.gov/pathogens]). We chose NARMS because it monitors enteric bacteria and pathogens across all states to determine their resistance to antimicrobials used in veterinary and human medicine. NARMS is a collaboration between agencies within the U.S. Department of Health and Human Services (HHS; Food and Drug Administration [FDA] and Centers for Disease Control and Prevention [CDC]) and the U.S. Department of Agriculture (USDA; Food Safety and Inspection Service, Animal and Plant Health Inspection Service, and the Agricultural Research Service). Each USDA agency taking part in NARMS tests bacterial samples at various stages of food animal production. The NARMS database holds bacterial genome sequences that originate from 15 distinct human, animal, and food sources and are categorized into four NARMS samples sources: (i) human clinical isolates; (ii) food animal production isolates from cecal samples at slaughter; (iii) samples routinely collected at inspected establishments as part of FSIS verification testing; and (iv) raw meats (chicken, ground turkey, ground beef, and pork chops) collected at retail outlets in 20 states (23 sites). We selected 837 chicken production isolates and 874 human clinical isolates, with collection dates from 1 January 2018 until 30 March 2020, using “species,” “host,” and “location” search filters (see Materials and Methods for a detailed description). E. coli isolate data were either available as Illumina or Oxford Nanopore FASTQ reads or as FASTA assemblies.

**FIG 1:**
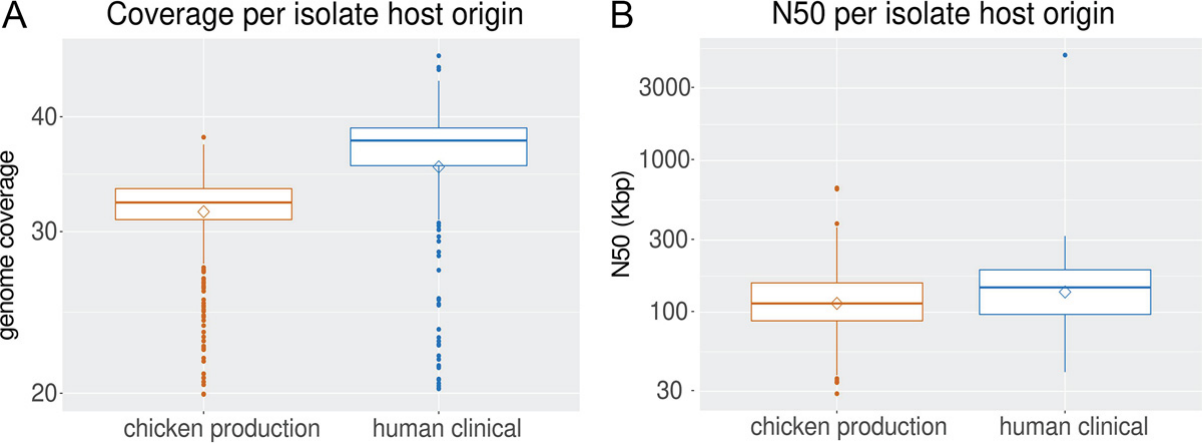
Average coverage, postnormalization, and average *N*_50_, postassembly, of human clinical and poultry production isolates. (A) Isolate FASTQ reads were downloaded from the NCBI and normalized to 30× using Trinity’s *insilico*_read_normalization.pl script. (B) Isolates with available FASTQ reads were assembled and annotated using Reads2Resistome, including *N*_50_ calculation by QUAST. FASTA assemblies were annotated using the additional script provided by Reads2Resistome for postassembly annotation. A diamond shape indicates a mean value, and the horizontal lines within a box indicate the mean. The upper and lower sides of the box correspond to the 25th and 75th percentiles. Plots were generated in R v4.0.4 using ggplot2 v3.3.3.

**FIG 2:**
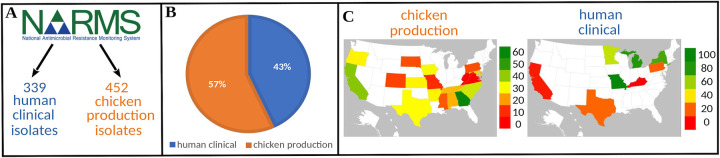
Graphical description of human clinical and poultry production isolates. (A and B) FASTQ reads or FASTA assemblies for each E. coli isolate was downloaded from the NARMS GenomeTrackr for chicken production and human clinical isolates. Isolates were selected from submission dates of 1 January 2018 to 30 March 2020 using the filtering criteria described in Materials and Methods. After quality control and filtering, 452 chicken production isolates (57%) and 399 human clinical isolates (43%) remained. (C) Isolate geographic locations across the United States. Maps were generated using the “Filled Map” chart type in Microsoft Excel from Microsoft Office 365 (Microsoft Corporation).

A total of 130 FASTA assemblies were obtained for human clinical isolates, whereas all chicken production isolates and remaining human clinical isolates had available FASTQ reads. The FASTA assemblies were obtained through the isolate’s associated BioProject, and quality control was done using QUAST (36). The FASTQ reads for each isolate were first normalized to 30× coverage using Trinity’s *insilico*_read_normalization.pl script (https://github.com/trinityrnaseq/trinityrnaseq/wiki). After quality control, the average genome coverage was 35.8× and 31.7× for human clinical and chicken production isolates, respectively (Fig. 1A). Totals of 385 chicken production isolates and 535 human clinical isolates were excluded from further analysis because they did not meet our preset quality control metrics based on genome coverage ≥20× and assembled genome quality (*N*_50_ ≥ 10,000 bp; Fig. 1B). The remaining E. coli isolates from chicken production and human clinical settings were confirmed to be E. coli through MLST (see Table S1, https://github.com/tseemann/mlst). This resulted in our study population consisting of 452 (57%) chicken production isolates and 339 (43%) human clinical isolates (Fig. 2A and B). Isolates originated from a diverse set of sources (see Table S1 in the supplemental material) and locations within the United States (Fig. 2C).

### Reads2Resistome enables high-throughput genome assembly and resistome characterization.

We developed Reads2Resistome (R2R) (37) to streamline the quality control, assembly, annotation, and resistome characterization of the chicken production and human clinical genomes (see the supplemental material for detailed description of Reads2Resistome [Fig. S1]). Utilizing a singularity (38) container, R2R maintains software and database versions resulting in reproducible results that can be replicated using the same input data and command-line options. We first assessed the accuracy and performance of R2R using short (Illumina) and long FASTQ reads (PacBio and Oxford Nanopore) of Salmonella enterica serovar Heidelberg and E. coli isolates recovered from the ceca of broiler chickens (see Table S2). Short and long reads-only assemblies resulted in the shortest run-time with an average of 6 min per sample, while hybrid assemblies (short and long reads were combined) required ~1 h per sample (see Table S3). Genomes assembled with short reads only or a hybrid approach had better resistome characterization and gene annotation than genomes assembled using long reads only (see Tables S4 and S5). For this study, all E. coli isolates were assembled from short Illumina reads except for a single isolate with long-read data (SRR11174251).

### AMR gene distribution not explained by isolation year or location.

We conducted principal-component analysis (PCA) to determine whether the presence or absence of identified AMR genes corresponded to the state origin of the isolates. Overlapping 95% confidence intervals were observed for all isolates in all states (see Fig. S1). We also conducted PCA on the presence/absence matrix of these AMR genes to assess impact of time of sampling. Separation of isolates based on their associated isolation year was also not observed (see Fig. S2). Accordingly, year and location will not be included in further analysis.

### Determining the phylogeny of *E. coli* isolate phylogroups.

**FIG 3:**
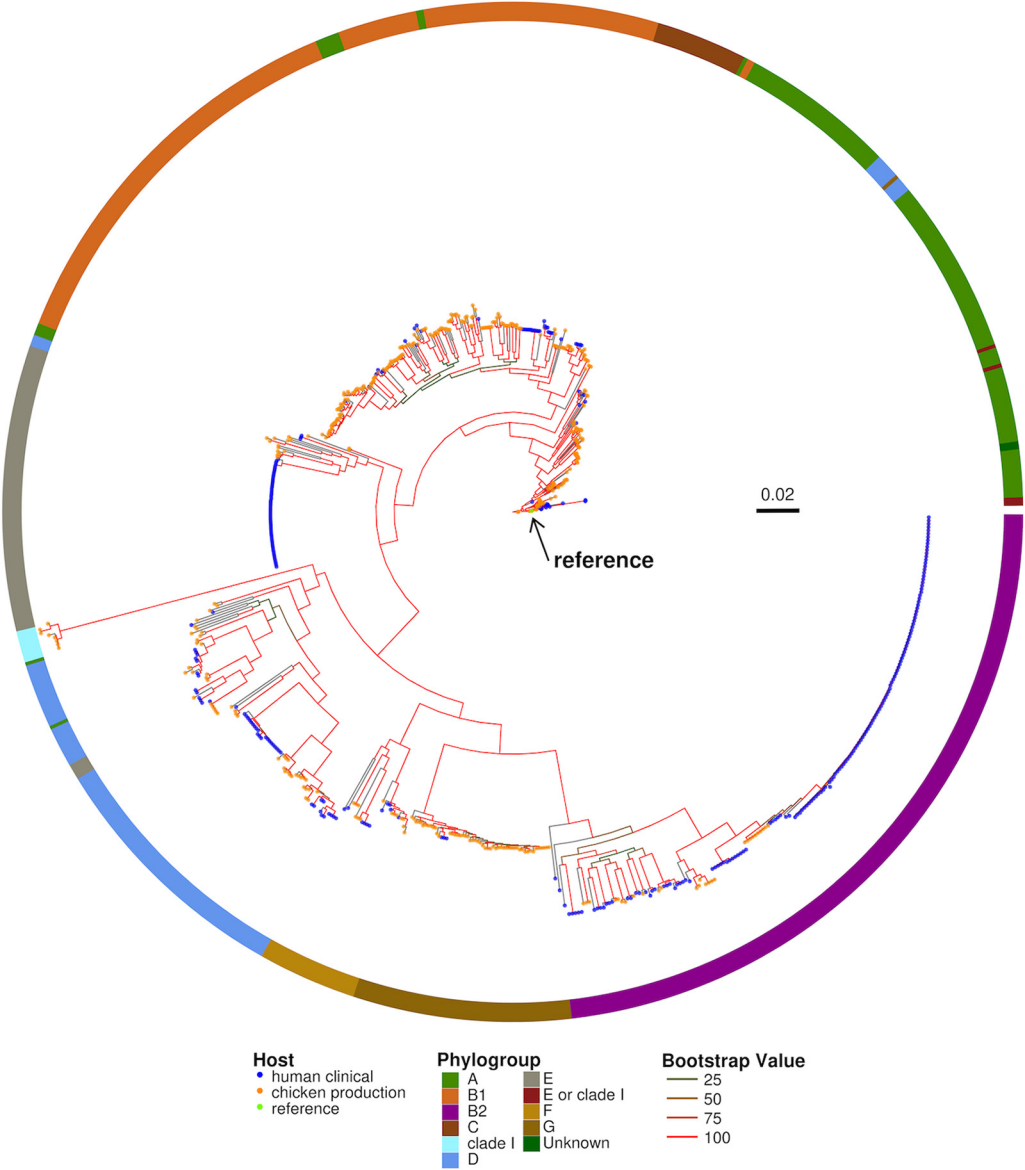
Whole-genome SNP-based maximum-likelihood phylogenetic tree. SNPs found between the E. coli K12-MG1655 (accession U00096) reference genome, and each of the 791 E. coli isolates was used for alignment using Snippy v4.6.0 (Snippy 2018). The resulting whole-genome SNP alignment was used to construct maximum-likelihood phylogenetic trees under a GTR+GAMMA model using RAxML with 100 bootstraps (RAxMLHPC-PTHREADS, v8.2.12). The strength of nodal support is indicated by the branch color: red (100), orange (50> to ≤ 75), dark orange (25> to ≤ 50), and green (0> to ≤ 25). (Ring) Phylogroups identified using ClermonTyping v1.4.0. Phylogroups are labeled by color: A (green), B1 (orange), B2 (magenta), C (brown), clade I (aquamarine), D (blue), E (gray), E or clade I (maroon), F (gold), G (dark gold), and unknown (dark green).

To determine whether there is an evolutionary based separation between chicken production and human clinical isolates, we performed a whole-genome alignment to the E. coli K12-MG1655 reference genome (accession U00096). Single-nucleotide polymorphisms (SNPs) with respect to the reference genome were used to generate a phylogenetic tree. In addition, we used ClermonTyping (39) to classify the isolates into phylogroups. E. coli isolates within the phylogenetic tree clustered into single clades and chicken production isolates were less divergent from the E. coli K-12 reference genome than human clinical isolates (Fig. 3). E. coli isolates were phylotyped into one of the phylogroups A, B1, B2, C, clade I, D, E, “E or clade I,” F, and G (Table 1). The majority of the E. coli genomes in our data set belonged to phylogroups A, B1, and B2. Phylogroups B1 and B2 were the most represented groups, with each accounting for ~23% of the total E. coli isolates. Phylogroup determination for one isolate was unsuccessful (identified as unknown), but the isolate clustered with phylogroup A isolates on the SNP-based tree.

**TABLE 1:** Distribution of phylogroups in chicken production and human clinical isolates^*a*^

Phylogroup	No. of isolates (% total)		
	Total (*n* = 791)	Chicken production (*n* = 452)	Human clinical (*n* = 339)
A	132 (16.62)	109 (85.58)*	23 (17.42)*
B1	183 (23.05)	148 (80.87)*	35 (19.13)*
B2	184 (23.17)	28 (15.21)*	156 (84.78)*
C	23 (2.90)	18 (78.26)	5 (21.74)
Clade I	8 (1.01)	8 (100)	0 (0)
D	103 (12.97)	56 (54.37)	47 (45.63)
E	75 (9.45)	14 (18.67)*	61 (81.33)*
E or clade I	4 (0.05)	4 (100)	0 (0)
F	26 (3.27)	15 (57.69)	11 (42.31)
G	55 (6.93)	52 (94.55)*	3 (5.45)*
Unknown	1 (0.13)	0 (0)	1 (100)

a The phylogroup for each isolate was determined using ClermonTyping v1.4.0 with default settings for each isolate genome assembly. Phylogroups that are significantly associated with a specific host are indicated with an asterisck (*), as determined by maximum-likelihood ratio testing with a Benjamini-Hochberg adjusted *P* value of <0.05.

Phylogroup classification is useful for the rapid and easy identification of potentially virulent and AMR isolates (39). Based on the Clermont quadruplex *in silico* method, our study isolates represented all seven main phylogroups (A, B1, B2, C, D, E, and F), cryptic clade I, and the newly identified phylogroup G. One isolate was classified as unknown. We found significant differences in the proportion of times phylogroups A, B1, B2, E, and G were seen with respect to their host origin. Phylogroups A, B1, and G were found at higher frequencies in chicken production isolates, while B2 and E were found at higher frequencies in human clinical isolates (Table 1). Eighty-one percent of chicken production isolates belonged to phylogroup B1, whereas 85% of human clinical isolates were classified as B2. The most divergent subclade within B2 consisted of closely related human clinical isolates, many of which originated from the same BioProject (PRJNA489090) which included many isolates from the human infant gut. Although the B2 phylogroup was dominated by human clinical isolates, there were a few chicken isolates nested within the B2 clades. Phylogroup E was dominated by closely related human clinical isolates (81%) and was the largest cluster of human clinical isolates within the phylogenetic tree. Isolates identified as belonging to the Escherichia cryptic clade I phylogroup (NCBI genome accession numbers SRR9984913, SRR10687720, SRR9852843, SRR9875260, SRR9875260, SRR10267809, SRR10687983, and SRR9852805) (40) clustered together within the phylogenetic tree and were all of chicken origin. No human clinical E. coli isolates were identified as belonging to clade I, even though clade I has been reported to have 2 to 3% prevalence in human E. coli isolates (41–43). Phylogroup G was dominated by chicken production isolates (95%).

The analysis presented above shows that in most cases the phylogroup classification supported the phylogenetic distribution within the SNP-based tree; however, we found several instances where human clinical and chicken production isolates were closely related. Likewise, we found cases where the SNP-based classification contradicted the phylogroup classification. For instance, human clinical strains mainly belong to phylogroup A (41–43); however, we identified 85.58% of our chicken production isolates belong to this phylogroup. Taken together, our isolate collection serves as a good representation and distribution of the currently described E. coli phylogroups, with a few exceptions.

### Chicken production and human clinical isolates harbor a diverse set of AMR genes and mutations.

**FIG 4:**
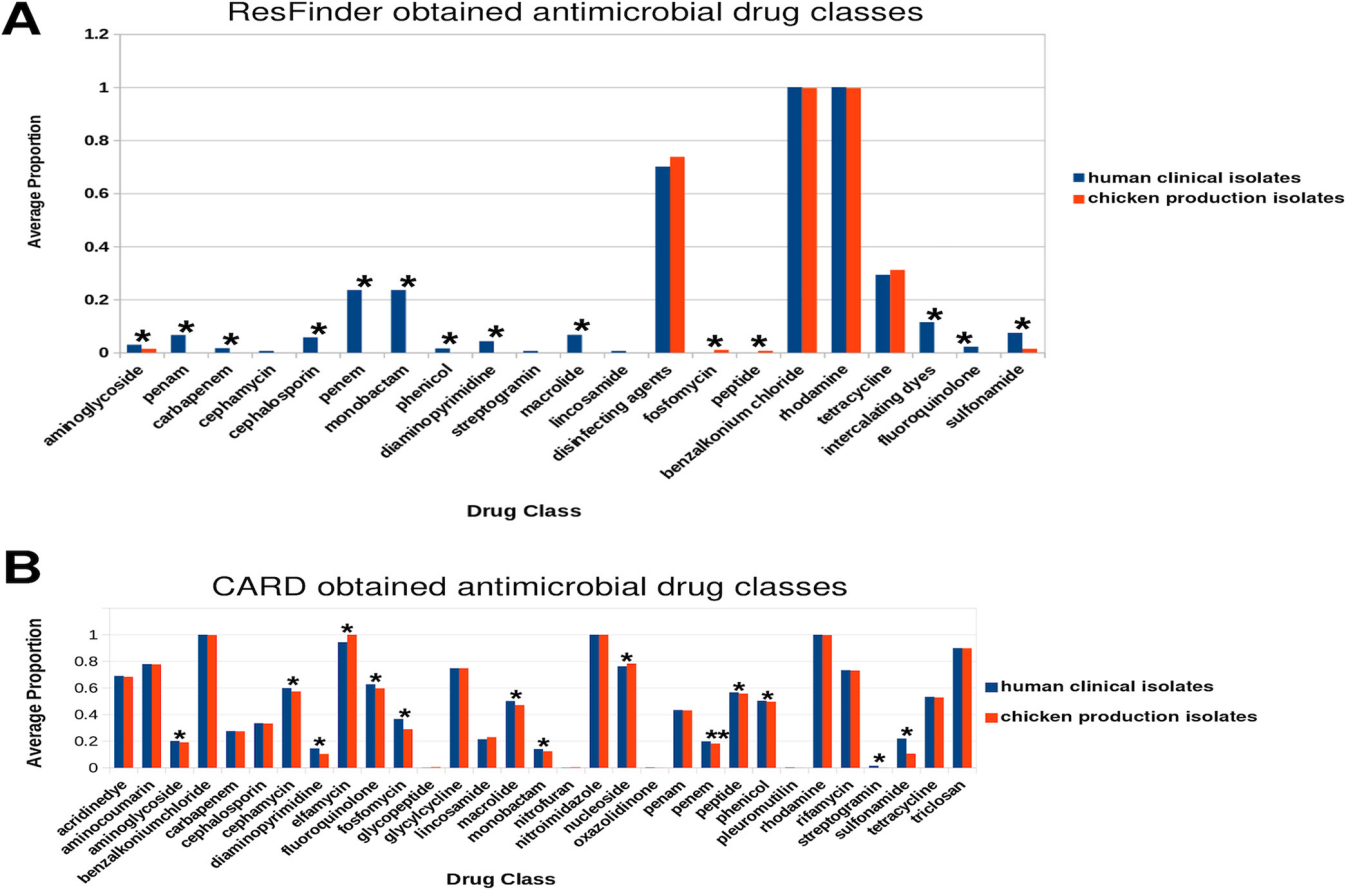
Average proportions of identified antimicrobial resistance genes in human clinical and poultry production isolates. (A) Acquired antimicrobial resistance genes identified within chicken production and human clinical isolate FASTA assemblies using ResFinder. ResFinder output hits for each gene were filtered (≥80% identity to database reference query). (B) Antimicrobial resistance drug classes identified within chicken production and human clinical isolate FASTA assemblies using Resistance Gene Identifier (RGI) and Abricate employed via Reads2Resistome. AMR database hits were filtered (≥95% identity to database reference query), and the highest hit from each database was retained. Genes conferring resistance to drug classes were enumerated for each isolate, and a proportion was calculated using the total number of genes in the study population conferring resistance to a given drug class. Drug classes significantly differing between chicken production isolates and human clinical isolates were determined by Wilcoxon rank sum test, and an asterisk (*) indicates a Benjamini-Hochberg adjusted *P* value of <0.05.

We used the ResFinder (44) to identify acquired AMR genes in each E. coli genome. In addition, we used R2R and the Comprehensive Antimicrobial Resistance Database (CARD) via the Resistance Gene Identifier (RGI) (45) (see Materials and Methods for details) to identify all AMR genes, including those encoded on the bacterial chromosome and gene mutations that can result in resistant genotypes. We found 42 acquired AMR genes predicted to confer resistance to 21 drug classes (Table 2). Fifty-seven percent of the isolates carried at least 1 acquired AMR gene, while one human clinical isolate carried 21 acquired AMR genes. Chicken production isolates were more likely to harbor acquired AMR genes (65.7%) than human clinical isolates (46.3%) (adjusted *P* ≪ 0). Chicken production E. coli isolates carried more acquired AMR genes per isolate (range, 0 to 8; mean, 1.97 per isolate) compared to human clinical isolates (range, 0 to 21; mean, 1.87 per isolate). Fosfomycin (*fosA7*) and peptide antibiotics (*mcr-9*) were significantly higher in chicken production isolates, while aminoglycosides [*aac(6′)-Ib-cr*] and fluoroquinolones (*qrnB19*) were significantly higher in human clinical isolates (Fig. 4A; see also Table S6).

**TABLE 2:** Acquired antimicrobial resistance genes identified within chicken production and human clinical isolate FASTA assemblies using ResFinder^*a*^

Gene	Drug class	Gene	Drug class
*aac(3)-IIa*	Aminoglycoside antibiotic	*dfrA1*	Diaminopyrimidine antibiotic
*aac(3)-IId*	Aminoglycoside antibiotic	*dfrA12*	Diaminopyrimidine antibiotic
*aac(3)-IV*	Aminoglycoside antibiotic	*dfrA14*	Diaminopyrimidine antibiotic
*aac(6′)-Ib-cr*	Fluoroquinolone antibiotic, Aminoglycoside antibiotic	*dfrA17*	Diaminopyrimidine antibiotic
*aadA1*	Aminoglycoside antibiotic	*erm*(*B*)	Lincosamide antibiotic, macrolide antibiotic, streptogramin antibiotic
*aadA2*	Aminoglycoside antibiotic	*floR*	Phenicol antibiotic
*aadA5*	Aminoglycoside antibiotic	*formA*	Disinfecting agents
*aph(3″)-Ib*	Aminoglycoside antibiotic	*fosA7*	Fosfomycin
*aph(3′)-Ia*	Aminoglycoside antibiotic	*mcr-9*	Peptide antibiotic
*aph(3′)-IIa*	Aminoglycoside antibiotic	*mdf*(*A*)	Rhodamine, tetracycline antibiotic, benzalkonium chloride
*aph(3′)-III*	Aminoglycoside antibiotic	*mph*(*A*)	Macrolide antibiotic
*aph(4)-Ia*	Aminoglycoside antibiotic	*qacE*	Disinfecting agents and intercalating dyes
*aph(6)-Ic*	Aminoglycoside antibiotic	*qnrB19*	Fluoroquinolone antibiotic
*aph(6)-Id*	Aminoglycoside antibiotic	*rmtB*	Aminoglycoside antibiotic
*bla*_CMY-2_	Penam, carbapenem, cephalosporin, cephamycin	*sitABCD*	Disinfecting agents
*bla*_CTX-M-14_	Cephalosporin	*sul1*	Sulfonamide antibiotic
*bla*_CTX-M-15_	Penam, cephalosporin	*sul2*	Sulfonamide antibiotic
*bla*_OXA-1_	Penam, carbapenem, cephalosporin	*sul3*	Sulfonamide antibiotic
*bla*_OXA-244_	Penam, carbapenem, cephalosporin	*tet*(*A*)	Tetracycline antibiotic
*bla*_TEM-1B_	Monobactam, penam, penem, cephalosporin	*tet*(*B*)	Tetracycline antibiotic
*catA1*	Phenicol antibiotic	*tet*(*C*)	Tetracycline antibiotic

a ResFinder output hits for each gene were filtered (≥80% identity to database reference query). The corresponding resistance-conferring drug class for each gene was identified using the Comprehensive Antibiotic Resistance Database (CARD).

**FIG 5:**
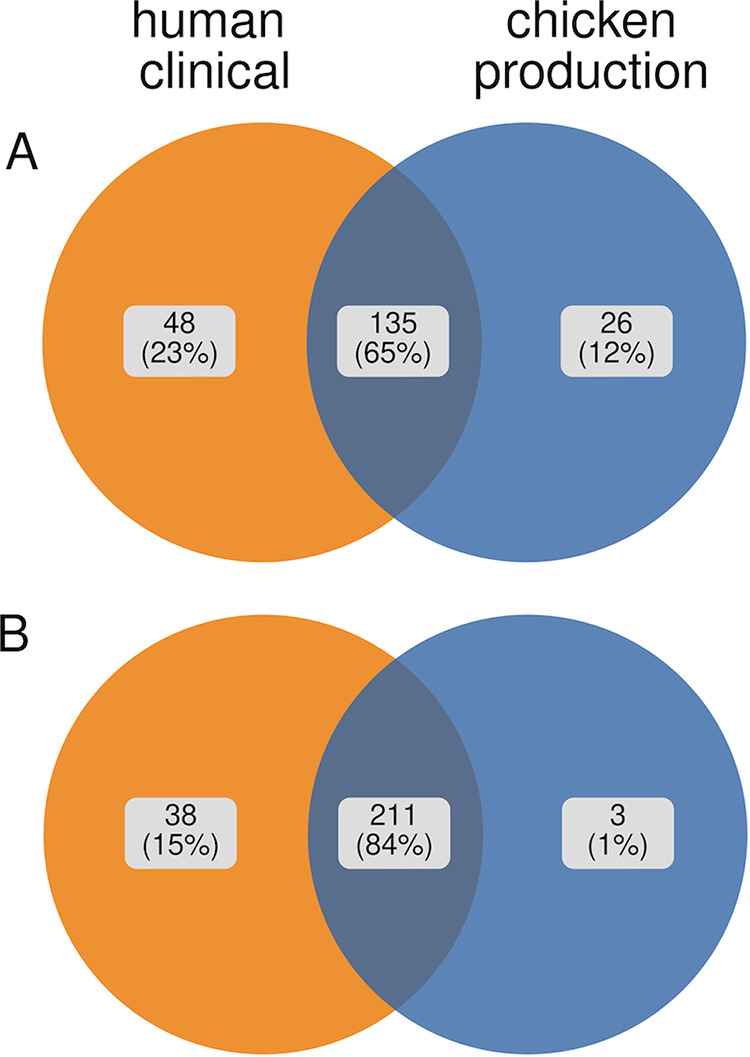
Venn diagrams depicting the percentage of antimicrobial resistance genes identified via Reads2Resistome (RGI) and The Comprehensive Antibiotic Resistance Database and ResFinder (A) and virulence factors identified via Reads2Resistome (Abricate) and the Virulence Factor Database (B).

Using the CARD database we found 208 AMR genes, including all but three genes [*aac(3′)-IIa*, *sitABCD*, and *formA*] identified by ResFinder (see Table S1). A majority of AMR genes were shared between human clinical isolates and chicken production isolates (Fig. 5A). The AMR genes were predicted to confer resistance to 31 drug classes, including cephalosporins, fluoroquinolones, penams, and tetracyclines (Fig. 4B). We found AMR genes for 31 drug classes in human clinical isolates, while 28 of the 31 drug classes were found in chicken production isolates. AMR genes absent from chicken production isolates were predicted to confer resistance to streptogramin (*ermB* and *msrA*), oxazolidinone (*msrA*), and pleuromutilin (*msrA* and *taeA*). We found significant differences in antimicrobial drug classes between chicken production and human clinical isolates for 14 of the 31 drug classes (adjusted *P* < 0.05, Fig. 4B; see also Table S7). Genes predicted to confer resistance to diaminopyrimidine, phenicol, sulfonamide, penem, aminoglycoside, fluoroquinolone, macrolide, and peptide antibiotics were more prevalent within human clinical isolates (adjusted *P* ≪ 0, Fig. 4B). Genes predicted to confer cephamycin resistance – a highly important antimicrobial group for human medicine (46) – were more prevalent in human clinical isolates (adjusted *P* ≪ 0, Fig. 4B) compared to chicken isolates. Genes predicted to confer resistance to elfamycin and nucleoside antibiotics were more prevalent within the chicken production isolates compared to human isolates (adjusted *P* ≪ 0, Fig. 4B). The frequency of genes predicted to confer resistance to tetracycline was similar (adjusted *P* = 0.27, Fig. 4B) between host origins.

It is important to mention that the majority of AMR genes identified by CARD are likely chromosomally encoded and may not be mobilizable. Many of the AMR genes harbored on the chromosome confer levels of resistance that are not medically relevant but may confer fitness advantages to these isolates in their native environments (47, 48). In contrast, most of the AMR genes identified by ResFinder are more likely to be mobilized within bacterial populations, via plasmids or other mobile genetic elements (MGEs), are therefore more likely to spread between/within E. coli strains originating from human and food animal populations. We hypothesized that a majority of the AMR genes identified by ResFinder were located either on plasmid or chromosomal contigs and not located in close proximity to other MGEs such as genomic islands, insertion sequences, gene cassettes, etc. This hypothesis was proposed due to all but three ResFinder identified AMR genes being also identified by CARD which are majorly present on chromosomal contigs. To test this hypothesis, we submitted four isolates to VRProfile2 (49), an online bacterial mobile element detection pipeline capable of predicting AMR gene presence on plasmid or chromosomal contigs or those in close proximity to other MGEs. VRProfile2 results indicated AMR genes found by ResFinder were located on plasmid contigs, as was the case for *aph(3′)-III*. No AMR genes were identified in close proximity to any other MGE types (see Table S8).

Overall, we observed a high prevalence of genes that can confer resistance to antimicrobials considered highly and critically important to human medicine in both chicken production and human clinical E. coli isolates. The prevalence of these genes in both human and chicken E. coli isolates indicates that these populations may serve as a possible reservoir of antimicrobial resistance.

### The presence of type III secretion system genes classified the *E. coli* isolates into two separate clusters.

**FIG 6:**
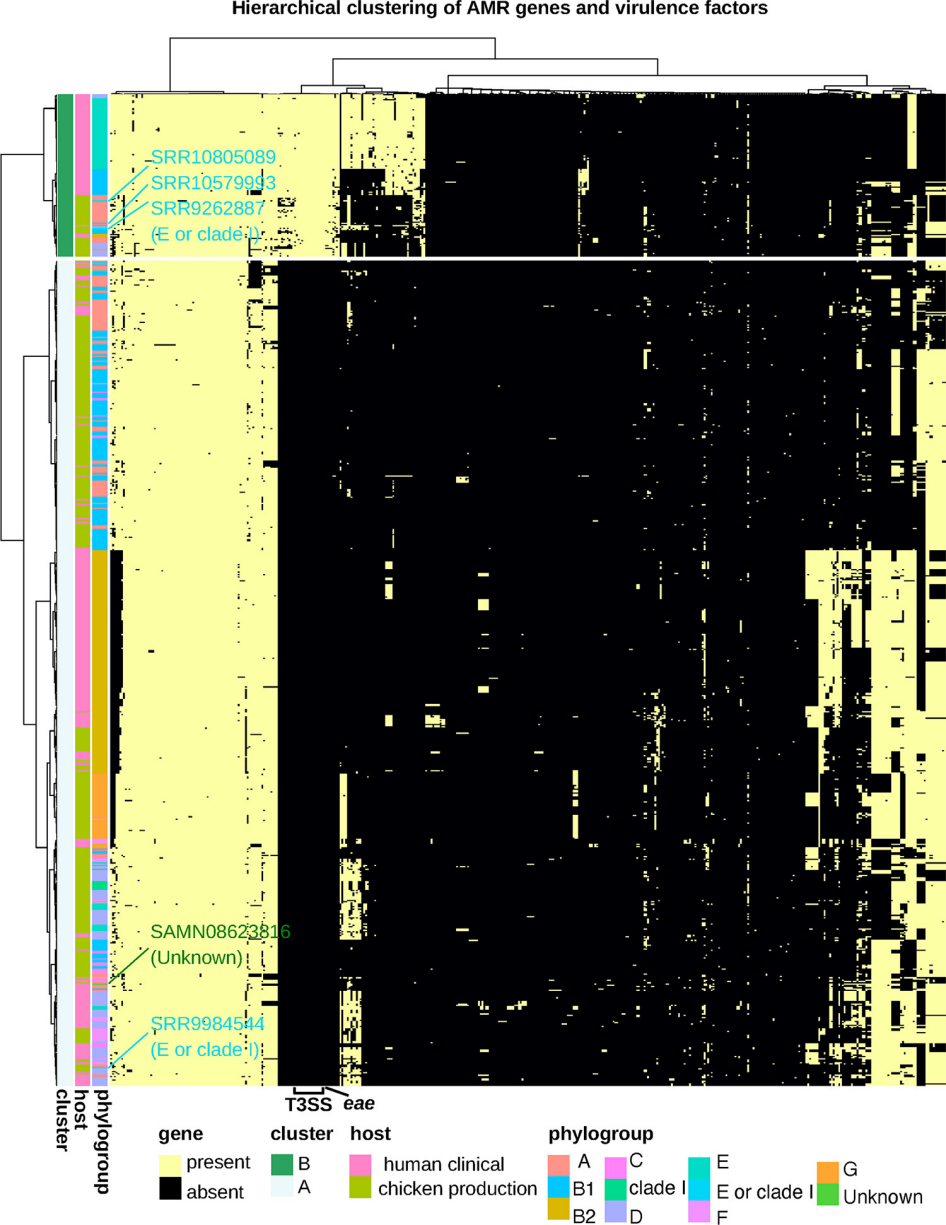
Heatmap of AMR genes and virulence factors across all isolates (see Table S1). Heatmap was generated in R v4.0.4 with pheatmap v1.0.12 (clustering_method = “average” [UPGMA], clustering_distance_cols = “binary,” clustering_distance_rows = “Euclidean”) using the filtered AMR gene and virulence factor table. Isolates from the phylogroups “unknown” and “E or clade I” are highlighted.

To determine whether AMR genes and VFs (Fig. 5B) could separate the human clinical and chicken production isolates into distinct groups, we employed hierarchical clustering. Hierarchical clustering was based on the filtered presence/absence table of AMR genes and VFs (Fig. 6; see also Table S1) and resulted in two distinct clusters (A and B) consisting of 661 and 130 isolates, respectively. These two clusters were significantly different from one another (adjusted *P = P* ≪  0.05) compared by isolate host origin. Cluster A comprised mostly of chicken production isolates (407 of 664 [61%]) while cluster B composed of human clinical isolates (85 of 130 [65%]).

**FIG 7:**
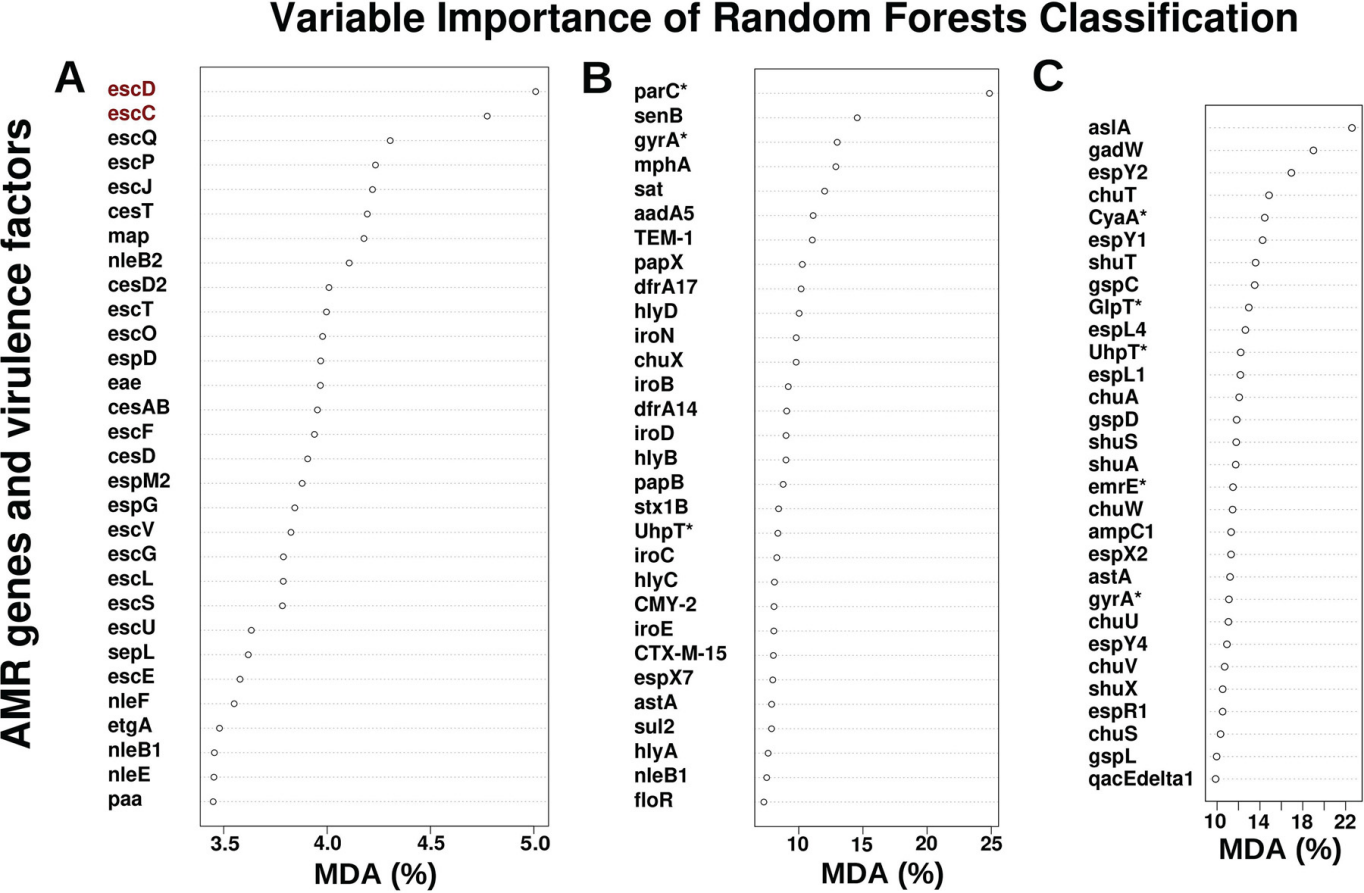
Mean decreasing accuracy (MDA) plot of variables in random forest classification models. MDA represents how much accuracy the model losses by exclusion of each variable (AMR gene or virulence factor). Random forest classification was performed on AMR gene and virulence factor table (see Materials and Methods for details). (A) Resulting MDA plot after classification of isolates into hierarchical clustering identified clusters A and B. Genes highlighted in red, on their own, result in 100% classification of isolates into hierarchical clustering identified clusters A and B. (B) Resulting MDA plot after classification of isolates into chicken production isolates and human clinical isolates. (C) Resulting MDA plot after classification of isolates into ClermonTyping identified phylogroups. Random forest classification in R was performed using the randomForest v4.6-14. Asterisks (*) indicate genes with mutations: *parC*(S80I), *gyrA*(S83L) (*n* = 30), *gyrA*(S84L) (*n* = 2), *gyrA*(D87Y) (*n* = 275), UtpT(E350Q), CyaA(S352T), and GlpT(E448K) (see Table S14).

To determine the genes driving the separation of these isolates into the two distinct clusters, we performed random forest classification on the filtered AMR gene and VF table (see Table S1). This reclassification resulted in 100% correct classification of all isolates to the two clusters (A and B) (see Table S9). Type 3 secretion system (T3SS) genes play a key role in the virulence of many Gram-negative bacterial pathogens (49) and were prevalent in all isolates from cluster B and completely absent from cluster A isolates. Twenty-five percent of all human clinical isolates (*n* = 339) carried genes for the T3SS operon (50), whereas only 9.29% of all chicken production isolates (*n* = 352) harbored the T3SS operon. Along with genes from the T3SS operon, the intimin gene (*eae*) that is required for intimate adherence and virulence in both humans and animals was a high contributor to the mean decreasing accuracy and was present in 100% of cluster B isolates but completely absent from cluster A isolates (Fig. 7A). Reducing the input AMR and virulence factors to only include the T3SS outer ring protein (*escD*) and the T3SS secretin (*escC*), two genes that encode oligomerizing proteins for the T3SS and that are essential for T3SS functionality (49), resulted in 100% correct classification of all isolates to either cluster A or B. Our results suggest that cluster B isolates that harbor T3SS may have the potential to deliver effector proteins that promote bacterial colonization, replication, and transmission in human host cell cytoplasm, while cluster A isolates may lack this capability. Nevertheless, we found both clusters contained isolates from human clinical and chicken production settings.

### AMR genes and virulence factors classified *E. coli* isolates by their host origin.

To assess which AMR genes and VFs differentiate isolates based on their host origin we performed Random Forests classification (Fig. 7B). This classification resulted in 1.1% (5 isolates) error in classifying chicken isolates and a 5.6% (19 isolates) error in classifying human clinical isolates (see Table S10). All misclassified isolates belonged to the hierarchical cluster A (see Table S11) described earlier. The E. coli topoisomerase IV (*parC*) gene with the S80I mutation, was the highest contributor to the mean deceasing accuracy. The *parC* gene plays a critical role in DNA replication and confers reduced susceptibility to fluoroquinolones if the relevant *parC* and gyrase A (*gyrA*) mutations are present together (47, 51–54). A total of 185 isolates (1 chicken production and 184 human clinical) had *parC* mutations, and 215 isolates (25 chicken production and 190 human clinical) had *gyrA* mutations. Four human clinical isolates had *parC* mutations but no *gyrA* mutation, while 34 isolates (24 chicken production and 10 human clinical) had *gyrA* mutations but no *parC* mutation. One E. coli isolate, misclassified as a human isolate, harbored both *parC* (S80I) and *gyrA* (S83L, S84L, and D87Y) mutations (see Table S12). The remaining 572 isolates did not have a *parC* or a *gyrA* mutation. In summary, *parC* and *gyrA* mutations that confer resistance to fluoroquinolones were present in many of the human clinical isolates, and it was a major factor that separated the E. coli isolates into their respective host origins.

The iron acquisition genes (*iroN*, *iroD*, and *iroE*) were high contributors to the mean decreasing accuracy of random forest classification. The catecholate siderophore uptake system (*iroBCDEN*) plays a critical role in virulence since iron is required for many cellular processes, but it is limited in host sites of extraintestinal infections (55). This gene cluster has been reported to be located in a pathogenicity island on the chromosome and has been found on plasmids (33, 56). However, this gene cluster was absent from all misclassified chicken isolates but was present in 56 and 7% of chicken production isolates and human clinical isolates, respectively (see Table S1). The enterotoxin TieB protein (*senB*) was the second most important factor and was absent from all chicken production isolates regardless of their random forest classification but present in 28% of human clinical isolates. Only one misclassified human clinical isolate harbored *senB*. These data suggest that the human and chicken isolates in our data set carry distinct AMR determinants and VFs which differentiate them from one another.

### AMR genes and VF content can classify *E. coli* isolates by phylogroup.

Next, we hypothesized that if the AMR genes and VF content is specific to each phylogroup, we should be able to classify the E. coli isolates correctly into their respective phylogroups based on their AMR and VF profiles. To evaluate this, we used random forests to classify the isolates into their identified phylogroups. Classification resulted in the following error rates: phylogroup A (11%), B1 (3%), B2 (0%), C (48%), clade I (0%), D (0%), E (5%), “E or clade I” (100%), F (4%), G (2%), and unknown (100%) (see Table S13). Unlike host origin classification, reducing the input table for phylogroup classification resulted in an increase in the misclassification error. Interestingly, the top 30 factors, needed to classify isolates into their respective phylogroups, consisted of 8 AMR genes and 22 VFs (Fig. 7C), suggesting that VFs have a greater influence than AMR genes for phylogroup classification.

Of 11 VF functions found using the virulence factor database (see Table S14), 6 were present in all phylogroups, and the remaining functions were either entirely absent from individual phylogroups or present in some (see Table S15). Interestingly, virulence functions relating to adherence, secretion systems, and iron uptake were present in all phylogroups, but functions relating to immune evasion were present only in phylogroups A, B1, B2, D, and E. (see Table S15). Thus, both chicken production and human clinical isolates have the potential to serve as VF reservoirs. Albeit E. coli isolates of phylogroups C, “E or clade I,” clade I, G and F, lacked many of the VF functions compared to A, B1, B2, D, and E.

We found that an isolate’s AMR and VF profile was sufficient, in most cases, to determine its phylogroup. In addition, misclassified isolates were supported by their AMR and VF similarity as seen using the UPGMA (unweighted pair-group method with arithmetic averages) clustering approach (see Materials and Methods for details; Fig. 6). For instance, the E. coli isolate that was identified by ClermonTyping as unknown was classified into phylogroup A based on its AMR gene and VF profile similarity with other phylogroup A, B1, and C isolates (Fig. 6). The misclassification of “E or clade I” isolates (NCBI accession numbers SRR10805089, SRR10579993, and SRR9262887) as phylogroups D and E, respectively, were also supported by clustering in close proximity to other phylogroup D and E isolates (Fig. 6). An additional “E or clade I” isolate (NCBI accession number SRR9984544) was misclassified as D but contained a similar AMR and VF profile as other phylogroup D isolates. The misclassification was due to the absence of iron uptake genes *shuS*, *shuA*, and *shuX*, as well as the non-locus of enterocyte effacement (LEE)-encoded T3SS genes *espL4* and *espY1*. Although the AMR and VFs harbored by an E. coli isolate provided sufficient signal for correct classification of phylogroups, it could not correctly classify all phylogroups identified by ClermonTyping. However, the misclassification of “unknown” groups and E or clade I isolates was explained and supported by their clustering based on the similarity of their AMR gene and VF profiles (Fig. 6). Similarly, the location of the “unknown” isolate within the phylogenetic tree supports the random forests classification (Fig. 3), i.e., clustered with phylogroup A isolates.

## DISCUSSION

In this study, we utilized E. coli isolates obtained from the NARMS surveillance program to study differences in phylotype, AMR, and VFs between chicken production and human clinical isolates. To do this, we first developed a massively parallel pipeline (Reads2Resistome) to streamline our genomic analysis and to ensure that our results are repeatable and reproducible. We found that E. coli can serve as a reservoir of AMR genes and VFs which supports previously reported findings (57–59). We determined that a majority of AMR genes and VFs identified were shared (65 and 84%, respectively) between human clinical isolates and chicken production isolates. Although there is a possibility of horizontal gene transfer in the environment, the E. coli isolates from this study were mostly host-specific, suggesting that strains from chicken production rarely colonize humans. We identified AMR genes that have been acquired, as well as those encoded on the chromosome that can acquire mutations that will result in reduced susceptibility to antibiotics. We confirmed the presence of 42 acquired AMR genes conferring resistance to 21 drug classes (Table 2, Fig. 4A, and Fig. 5A). The drug classes identified include those considered critically and highly important to human medicine. For example, genes conferring resistance to fosfomycin, considered a high priority and critically important antimicrobial by the WHO (46), were significantly more prevalent within the chicken production isolates. Utilizing the CARD database, a collection of all AMR genes, including those with mutations known to confer a level of resistance to known antimicrobials, we identified genes conferring resistance to 31 antibiotic drug classes in our human clinical isolates, 28 of which were observed in the chicken production isolates (Fig. 4B). In addition, genes conferring resistance to these 31 drug classes were observed across a majority of identified phylogroups.

We determined that chicken production isolates carried a higher proportion of acquired AMR genes than human clinical isolates. Acquired AMR genes are those which have been obtained by a strain via mobile genetic elements such as plasmids, integrative conjugative elements, bacteriophages, insertion sequences, and gene capture elements such as a site-specific recombination system (59). Acquired AMR genes are important in the context of animal-to-human transmission since they are more readily shared between bacteria and provide a route for dissemination of AMR genes. The high proportion of benzalkonium chloride is not surprising due to its high usage, as a disinfectant, in the food and medical industries (60). The gene conferring resistance to benzalkonium chloride, *mdfA*, a multidrug efflux system, also confers resistance to tetracycline and rhodamine. When comparing the presence of AMR genes from CARD, which includes genes with mutations resulting in a resistance genotype to acquired resistance genes from ResFinder, we observed a drastic decline in the number of drug classes a given isolate may present resistance to. The prevalence of tetracycline in human clinical and chicken production isolates is expected due to its widespread usage in both agriculture and human clinical medicine and is of concern because of its high importance to human medicine.

Of particular interest is our finding of widespread potential resistance to fluoroquinolone antibiotics within the chicken production isolates since their usage was banned in poultry in 2005 (61), especially given that our data were obtained from samples between 2018 and 2020. Fluoroquinolone is a critically important antimicrobial drug class to human medicine due to its use in treatment of Campylobacter spp., invasive Salmonella spp., and multidrug-resistant *Shigella* spp. infections (46). Further investigation of the causes for fluoroquinolone resistance persistence in poultry is needed to ensure its efficacy in treating human infections. Taken together, we identified many antimicrobial drug classes which were highly present, but expected, in our study population. However, the presence of AMR genes conferring resistance to antibiotics banned for use in poultry, or food animal production in general, raises concern for their future effectiveness in treating human infections and diseases.

We found 11 virulence functions, conferred by 251 identified VFs (Fig. 5B; see also Table S14) to be present in both chicken and human clinical isolates. Based on hierarchical clustering of T3SS genes, the E. coli isolates were classified into two distinct clusters, A and B. The T3SS is associated with pathogens which can adhere to the epithelial surface of the host, many of which can cause diarrheal disease; the second leading cause of death in children globally (62, 63). Cluster A contained a higher representation of chicken production isolates, while cluster B comprised mainly of human clinical isolates. This result suggests that E. coli isolates from chicken production and human clinical isolates differ in their carriage of T3SS genes. The T3SS is essential for virulence and colonization of the human gut and is the genetic basis for enteropathogenic E. coli classification (63). In addition, the T3SS in poultry contributes to virulence in avian-pathogenic E. coli (64). This result suggests that the T3SS genes can be used as targets for a rapid on-farm and clinical diagnostic detection of virulent E. coli strains in chicken production and hospital settings. Identifying a lower proportion of chicken production isolates harboring the T3SS is a positive finding since the T3SS increases virulence in both humans and chickens.

Classification into host origin was successful with little error. Two of the most important factors for this separation were the *parC* gene (S80I) and *gyrA* (S83L [*n* = 30], S84L [*n* = 2], and D87Y [*n* = 275]), which are both linked to fluoroquinolone resistance. It has been documented that these resistance mutants are commonly found in environmental E. coli even in the absence of fluoroquinolone selective pressures (52). We speculate that these genes, and their respective mutations, which relate to DNA replication and transcription, may confer a fitness advantage. The secreted enterotoxin TieB, encoded by *senB*, was the second most important factor in separating human and chicken isolates. *senB* was only identified in human clinical isolates, and it was expected since *senB* is typically associated with enteroinvasive and uropathogenic E. coli infection in humans (65). Similarly, *TEM-1*, a broad-spectrum beta-lactamase conferring resistance to penicillins and first-generation cephalosporins, was also a high contributor to the separation of hosts and was only present in human-linked isolates. While *TEM-1* has been identified in poultry isolates (66), cephalosporin usage is more common in beef production (67).

This study reemphasizes the utility of phylogroup classification as a convenient way to identify pathogenic strains. The most recent version of ClermonTyper (39) relies on five genes (*arpA*, *chuA*, yj*aA*, TspE4.C2, and *ybgD*) for phylogroup classification, only one of the quadruplex genes, *chuA* (outer membrane protein responsible for heme uptake), was present in our presence/absence table of identified AMR and VFs. Even though *yjaA* (gene that differentiates phylogroups B2 and D isolates) was absent from the AMR gene and VF table, we were able to reclassify B2 and D isolates with 100% accuracy. Similarly, TspE4.C2 gene differentiates phylogroup A from phylogroup B1, and its absence from our AMR gene and VF table resulted in an 11% classification error for A isolates and a 3% error for B1 isolates. The absence of four of the five ClermonTyping genes might have contributed to the 48% error in classifying phylogroup C. The ability to separate phylogroups F and G from B2 was recently accomplished with the addition of the genes *cfaB* (CFA/I fimbrial subunit B), specific to phylogroup G strains, and *ybgD* (fimbrial-like adhesin protein), specific to phylogroup F strains (39, 68). The absence of these genes in our table only resulted in one phylogroup F (4% error rate) and one phylogroup G misclassified isolates (2% error rate). Our results show that the phylotype classification based on AMR and VFs could be achieved even when the majority of the genes used for Clermont E. coli phylotyping are missing.

The use of whole-genome sequencing (WGS) is an effective tool to predict the AMR and virulence potential of a bacterium; however, WGS still has its limitations. The depth of coverage is crucial for genome assembly and gene annotation, both of which can be significantly influenced by a coverage of less than 30×. Our study excluded many isolates from both chicken production and the human clinical settings due to low sequencing coverage. Due to the nature of the database chosen for isolate selection, i.e., a surveillance system for foodborne and other enteric bacteria, we expected an over representation of isolates harboring AMR genes and VFs, and an under-representation of susceptible commensal and environmental E. coli isolates in our data sets.

In addition, the choice of AMR and virulence databases is important as not all databases have the same entries and methods of detection. Here, we implemented Reads2Resistome which allowed us to incorporate the results from multiple databases. Our final AMR gene and VFs were comprised of the highest percent hit based on similarity to the reference gene (cutoff for gene coverage >80%) from each database. Another limitation of this study is the lack of AMR or virulence phenotype data for each isolate. Therefore, the presence an AMR gene or VFs in a genome is not a confirmation that the isolate is resistant to the antibiotic/drug class predicted or that the isolate can cause diseases.

## MATERIALS AND METHODS

### Study isolates, normalization, and assembly.

A total of 1,711 Escherichia coli isolates, including 874 human clinical and 837 chicken production isolates, were selected from the USDA’s GenomeTrackr network via NCBI’s Pathogen Detection Network. Agricultural poultry isolates were selected based on the following criteria: species, “*E. coli* and *Shigella*”; location, “USA”; target creation, “01/01/2018 to 03/30/2020”; host, “Poultry,” “poultry,” “Gallus gallus,” and “Gallus gallus *domesticus*.” Human clinical isolates were selected based on the following criteria: species, “*E. coli* and *Shigella*”; location, “USA”; target creation, “01/01/2018 to 03/30/2020”; host, “Homo sapiens,” “*Homosapiens*,” “homo sapiens,” “human,” “human (infant).” Only isolates with a “strain” denoted as “E. coli” were retained for downstream analysis; isolates identified as “*Shigella*” were not retained. If available, Sequence Read Archive (SRA) reads, in FASTQ format, were downloaded based on the BioSample ID for both agricultural and human isolates. In the event the SRA reads were not available, FASTA assemblies were downloaded from the associated BioProject submission. To ensure accurate and unbiased antimicrobial resistance identification, SRA reads were further filtered based on coverage ≥20×. To guarantee inclusion of quality genome assemblies, FASTA assemblies were filtered based on having an *N*_50_ greater than 10,000 bp. Three isolates in total were identified to be identical and were removed from downstream statistical analysis.

To compare genetic structure and contents, specifically AMR genes, virulence factors, and plasmid replicons across our study isolates, we assembled the SRA FASTQ reads into genomes, annotated these genomes, and annotated the downloaded FASTA assemblies. To reduce bias in gene identification and assembly quality across isolates, SRA reads were normalized to 30× coverage using the *insilico*_read_normalization.pl script from Trinity RNA-Seq v2.11.0 (Trinity’s GitHub). SRA reads were assembled using Reads2Resistome v0.0.2 (options: slidingwindow = 4:20, assembly = nonhybrid, threads 60). Resulting SRA assemblies were further filtered based on the *N*_50_ (>10,000 bp) and final coverage (≥20×) like the FASTA assemblies described earlier. The final study data set consisted of R2R assembled isolate genomes and downloaded FASTA assemblies totaling 791 E. coli isolates (452 chicken production and 339 human clinical; Fig. 2A).

### Phylogenetic and sequence type analysis.

To evaluate the relatedness of the isolates with respect to their assembled genomes, we constructed a phylogenetic tree utilizing SNPs. These SNPs were identified by comparing the E. coli K12-MG1655 (accession U00096) reference genome to each of the 791 E. coli isolates using Snippy v4.6.0 (69). The resulting whole-genome SNP alignment was then used to construct a maximum-likelihood phylogenetic tree using the GTR+GAMMA model of evolution and utilizing RAxML (RAxMLHPC-PTHREADS) v8.2.12 (70, 71). In addition, we performed 100 bootstraps to assess nodal support. Only 100 bootstraps were performed due to time and computational limitations; it took 75.49 days utilizing 64 compute cores and 504 Gb memory to construct the phylogenetic tree and perform the 100 bootstraps. To determine which of the seven main phylogroups the isolates belong to, we used ClermonTyping v1.4.0 (39), utilizing the default settings, to determine the phylogroup designation (A, B1, B2, C, D, E, and F) for each isolate. Tree visualization, along with phylogroup and metadata overlay, was done in R v4.0.4 using ggtree v2.4.1 (72), factoextra v1.0.7 (73), and ggnewscale v0.4.5 (74). The sequence type of each isolate was determined using ECTyper v1.0.0 (75) with default settings.

### Antimicrobial resistance gene and virulence-factor characterization using Reads2Resistome.

Reads2Resistome (R2R; see the supplemental Materials and Methods) was used to characterize the antimicrobial resistance genes, virulence factors, and prophage regions. Reads2Resistome utilizes ABRICATE v0.5 (76) to screen the assembled contigs against the following databases via BLAST and reports a percentage identity to the reference sequence. Databases used by ABRICATE, and provided by R2R, were as follows: the ARG-ANNOT antibiotic resistance gene database (77), the Comprehensive Antibiotic Resistance Database (CARD) (45), the MEGARes Antimicrobial Database for High-Throughput Sequencing (78), NCBI AMRFinderPlus (79), PlasmidFinder (80), and the VirulenceFinder database (81). Downloaded FASTA assemblies’ resistome characterization was performed by the modified Reads2Resistome (R2R-0.0.1-Fasta-QC-Ann-Only.nf). In addition, resistance gene identification was performed using the Resistance Gene Identifier v5.1.1 (45) (RGI), and acquired resistance genes were identified using ResFinder (82). AMR genes and virulence factors with the highest percent identity across all databases were selected for further analysis. The heatmap of AMR genes and virulence factors, using the table (described below; see also Table S1) was generated using pheatmap v1.0.12 in R v4.0.4 with the “average” (unweighted pair group method with arithmetic mean [UPGMA]) clustering method (see Fig. S1) (83). (See the supplemental Materials and Methods for further description of R2R). Mobile genetic elements were determined for selected isolates using VRProfile2 (updated most recently on 2 October 2021) (49).

### Hierarchical clustering and statistical analysis of resistome and virulence factors.

To evaluate the relationships between poultry and human isolates with respect to their AMR and virulence factors, we employed hierarchical clustering and ordination analysis. AMR and virulence database hits from R2R and RGI were filtered utilizing ≥95% and ≥80% sequence identities to the reference database query, respectively. ResFinder results were filtered utilizing ≥85% identity to the reference database query. A table was then generated based on the presence/absence of identified VFs and AMR genes from all isolates (see Table S1). A distance matrix was generated using the tanimoto metric via the Distance() (84) function from the IntClust v0.1.0 (85) package. hclust() from the stats v3.6.2 package (86) was then utilized to perform the hierarchical clustering under the “average” (UPGMA) method using the determined optimal number of clusters. We identified the optimal number of clusters which separate the 791 isolates using the silhouette method implemented by the fviz_nbclust() function from the factoextra v1.0.7 package (73). Drug classes associated with each identified AMR gene were determined using the output from RGI, in combination with the CARD database (45), and virulence factor-associated functions were determined using the comparative tables from the Virulence Factor Database (81). Genes conferring resistance to drug classes were enumerated for each isolate, and a proportion was calculated using the total number of genes in the study population conferring resistance to a given drug class. Drug classes significantly differing between hosts were determined by using a Wilcoxon rank sum test with the wilcox.test() function from the stats v3.6.2 package (86). *P* value adjustment for multiple comparisons was conducted using the p.adjust() function, according to the Benjamini and Hochberg (“BH”) method, from the stats v3.6.2 package (86). PCA was conducted using the prcomp() function from the stats v3.6.2 package (86), and plots were generated using ggplot2 (87). All analyses were done in R v4.0.4 utilizing RStudio v1.2.1106 (88).

### Random forest classification.

To determine the most influential AMR and virulence factors driving the separation of the isolates into (i) clusters identified via hierarchical clustering, (ii) isolate host origin, and (iii) identified phylogroups, we employed random forest classification (89). The described presence/absence table of AMR genes and virulence factors was used as input (see Table S1). TuneRF() (90) was used to determine the optimal mtry parameter value, with respect to out-of-bag error estimate, for RandomForest(). RandomForest() v4.6-14 (90) was used for classification with nttree = 200 based on the filtered AMR and virulence-factor presence/absence table. All plots were generated using ggplot2 v3.3.3 (87). All analyses were done in R v4.0.4 utilizing RStudio v1.2.1106 (88).

### Data availability.

All raw isolate data, both FASTQ and FASTA files, were obtained from and are available through the NCBI using the NCBI accession numbers in Table S1 in the supplemental material. All other data are available upon request.
