# Supplementary material for: Chicken Production and Human Clinical Escherichia coli Isolates Differ in Their Carriage of Antimicrobial Resistance and Virulence Factors
Source: Appl Environ Microbiol. 2023 Jan 18;89(2):e01167-22. doi: 10.1128/aem.01167-22 (PMC9973021; doi:10.1128/aem.01167-22)
Supplement: Supplemental file 1 — Supplemental text, Fig. S1 to S3, and Tables S2 to S7 and S9 to S15. Download aem.01167-22-s0001.pdf, PDF file, 0.9 MB [file aem.01167-22-s0001.pdf]

Supplementary Data for Manuscript

Chicken production and human clinical *Escherichia coli* isolates differ in their carriage of antimicrobial resistance and virulence factors

Reed Woyda[1,*], Adelumola Oladeinde[2,*], Zaid Abdo[1,3,*]

[1]Program of Cell and Molecular Biology, Colorado State University, Fort Collins, Colorado, USA

[2]U.S. National Poultry Research Center, USDA-ARS, Athens, Georgia, USA

[3]Department of Microbiology, Immunology and Pathology, Colorado State University, Fort Collins, Colorado, USA

1) **Supplementary Materials and Methods**
2) **Supplementary Results**
3) **Supplementary Figures**
4) **Supplementary Tables**
5) **Supplementary References**

1) Supplemental Materials and Methods

Reads2Resistome: An adaptable and high-throughput whole-genome sequencing pipeline for bacterial resistome characterization

Reads2Resistome is scripted using Nextflow [1] a parallel Domain Specific Languages (DSL) workflow framework, and is integrated with Singularity [2], an open-source container platform with focus towards high-performance computing (HPC) workloads. The Reads2Resistome pipeline includes three main steps: quality control, assembly, and annotation of assembled bacterial contigs. Reads2Resistome takes long read sequences, short read sequences or both as input and performs quality control of short reads using Trimmomatic [3] and long read quality visualization using NanoPlot [4]. Both quality-controlled short reads and long reads are then assembled using Unicycler [5]. Unicycler generates an assembly graph using short reads, then uses long reads to simplify the graph to generate accurate assemblies. In the event the input consists of short reads only, Unicycler employs SPAdes [6] for assembly and subsequently polishes the resulting graph by bridging contigs. Long read-only assembly is performed using miniasm [7] and Racon [8] employed through Unicycler. Annotation is performed with Prokka [9] using one of the provided custom databases (described in the Resistome characterization section below), which are pre-built from collections of specific bacterial species and subtypes, or using the Prokka default database. Resistome characterization is performed using ABRICATE [10]. Nextflow implementation using Singularity provides version control over the various open-source tools ensuring reproducible results. Reads2Resistome output contains the following for each input isolate: visualization of both raw and quality-controlled reads; assembled contigs with a corresponding assembly graph along with an assembly quality assessment; gene and resistome annotation files; genome alignment files in BAM format; and optional serovar predictions. All documentation and pipeline usage are publicly available at https://github.com/BioRRW/Reads2Resistome.

Streamline high-throughput analysis

Reads2Resistome is designed for high-throughput bacterial sequence input and performs quality control, genome assembly and subsequent genome and AMR and virulence gene annotation in a parallel, high-throughput manner (**Figure S3**). Reads2Resistome is able to accommodate input of different species within the

same run and can perform species-specific genome assembly quality control and gene annotation. A comma-separated values (CSV) file, generated by the user, enables input of multiple different isolates regardless of the isolate identity. The user can also specify a pipeline-provided database for genome annotation or can choose to use the default database utilized by Prokka. For genome assembly quality assessment, done by QUAST, the user can optionally add a user-provided reference genome for additional reference-specific metrics. Pipeline outputs for quality control and genome quality assessment are aggregated by MultiQC [11] into a HTML report. In addition to quality control, assembly and annotation, genome alignments are generated for further comparison. For *Salmonella spp.* optional serovar prediction is performed using SISTR [12].

Adaptable to cutting-edge sequencing technologies

Inclusion of long read sequences into bacteria assembly aids in resolving repeat regions of genomes and contributes to genome completeness [13]. Reads2Resistome is designed to be adaptable and flexible in that it can accommodate assembly in three different approaches: long read-only assembly, short read-only assembly, and hybrid assembly. In each assembly approach, Unicycler is used to generate genome assemblies and assembly graphs which are visualized with Bandage [14].

Resistome characterization

Reads2Resistome characterizes resistome content using ABRICATE [10] and Phigaro [15]. ABRICATE uses the assembled contigs to screen for AMR and virulence genes from various databases; ARG-ANNOT antibiotic resistance gene database [16] the Comprehensive Antibiotic Resistance Database (CARD) [17], MEGARes Antimicrobial Database for High-Throughput Sequencing [18], NCBI AMRFinderPlus [19], PlasmidFinder [20], ResFinder [21] and VirulenceFinder database [22]. ABRICATE compiles results into a single report containing hits from each database and Reads2Resistome provides an output file for each isolate. Phigaro uses the assembled contigs to detect putative taxonomic annotations and the output is collected and reported by Reads2Resistome for each isolate.

2) Supplemental Results

Reads2Resistome assessment

Using Reads2Resistome we assembled and characterized the AMR genes, virulence genes and prophage sequences of genomes associated with two bacterial isolates recovered from the ceca of 2-week-old broiler chickens; SH-IC: *Salmonella enterica* serovar Heidelberg (S. Heidelberg) and EC-IC: *Escherichia coli* (**Table S2**). Illumina, PacBio and Oxford Nanopore MinION sequences were used to evaluate and compare the three assembly methods available through the pipeline: short read-only, long read-only and hybrid assembly.

Short and long read-only assemblies, regardless of the read source, resulted in the shortest run-time with an average of 6 minutes per sample. Hybrid assembly, as expected, was the most time-intensive assembly method taking on average 1 hour and 8 minutes per sample, regardless of long read source (**Table S3**).

Genome assembly and annotation metrics were compiled from QUAST and Prokka outputs. Hybrid assembly of both EC-IC and SH-IC using MinION long reads and Illumina short reads gave the fewest contigs, longest total length and highest number of annotated genes as compared to long read assembly using MinION. Hybrid assembly of both isolates using PacBio reads resulted in fewer contigs but comparable total length to that of the MinION hybrid assembly. Genome contiguity was best obtained by hybrid assembly and can be visualized with Bandage-generated graphs. While hybrid and long read-only assemblies are comparable with respect to number of contigs and genome length, the long read-only assembly greatly lacked in annotated genomic features and resistome elements.

Annotated genes and features across all assembly methods for both isolates were considerably reduced under the long read-only assembly, while both short read and hybrid methods resulted in comparable numbers of annotated genes. We suspect this is due to relative lower quality of long reads as compared to Illumina short reads. This is mirrored in AMR and virulence gene characterization and prophage identification. While both short read and hybrid assembly methods for both isolates resulted in comparable identified resistome elements and prophage sequences, long read-only assembly identified elements were significantly reduced (**Table S4, Table S5**).

The pipeline was run using the following commands for short read-only, hybrid, and long read-only assemblies, respectively:

```
$ nextflow R2R-0.0.2.nf --assembly nonhybrid--input containers/data/input_nonhybrid.csv --output temp/output -w temp/work --threads 64  -with-report --name R2R_Nonhybrid_Assembly
```

```
$ nextflow R2R-0.0.2.nf  --input containers/data/input_hybrid.csv --output temp/output -w temp/work --threads 64 -with-report --name R2R_Hybrid_Assembly
```

```
$ nextflow R2R-0.0.2.nf --assembly longread --input containers/data/input_longread.csv --output temp/output -w temp/work --threads 64 -with-report --name R2R_Long-Read_Assembly
```

Each command was executed independently on a Linux server with 128 compute cores and 504GB of memory. Resources allocated and run-time in Table 2 were obtained from the "report.html" which is generated using the '-with-report' option.

Results from our case study indicated that a highly contiguous genome assembly with robust gene annotation, prophage identification, and resistome characterization is best obtained under a hybrid assembly approach. While hybrid assembly is the most time-intensive assembly method, it produces the most complete annotated genomes in our case study. Long read-only assembly is able to produce a respectable genome length with high contiguity but falls short when annotating genomic features.

3) Supplementary Figures

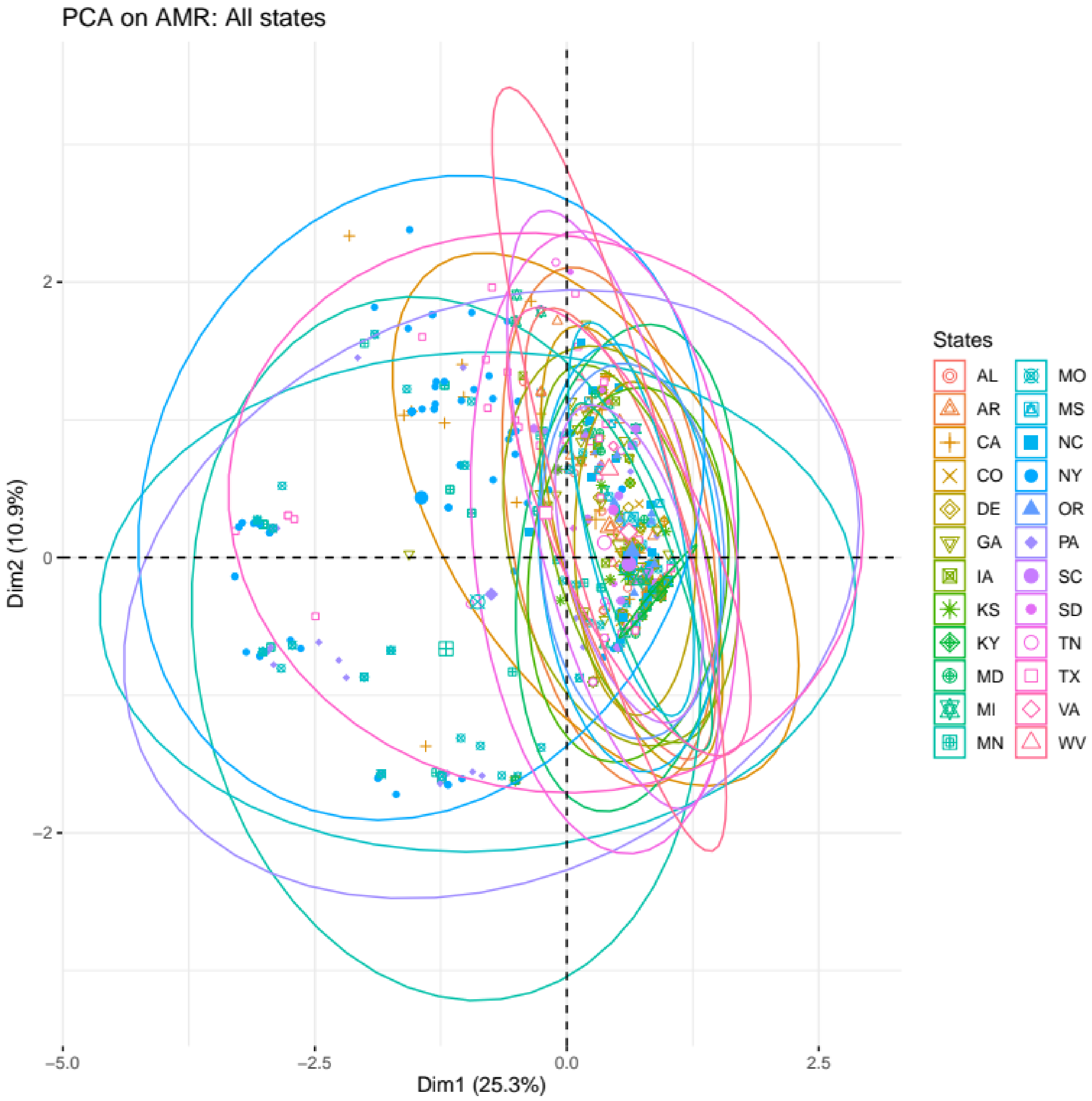

Figure S1. Principal component analysis of identified antimicrobial resistance genes from RGI and ResFinder. Isolates are labeled corresponding to their state origin.

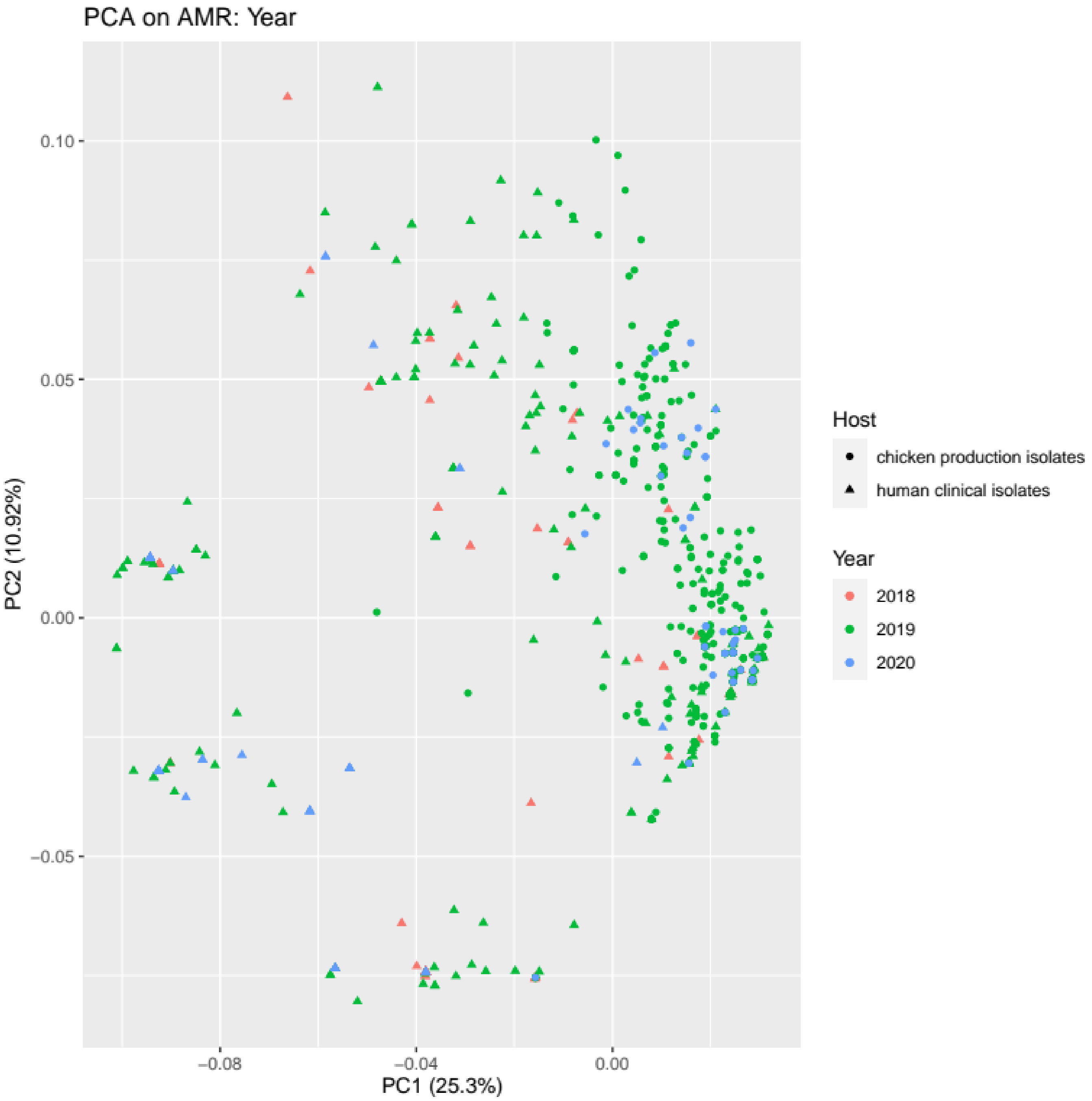

Figure S2. Principal component analysis of identified antimicrobial resistance genes from RGI and ResFinder. Isolates are labeled with the corresponding sampling year.

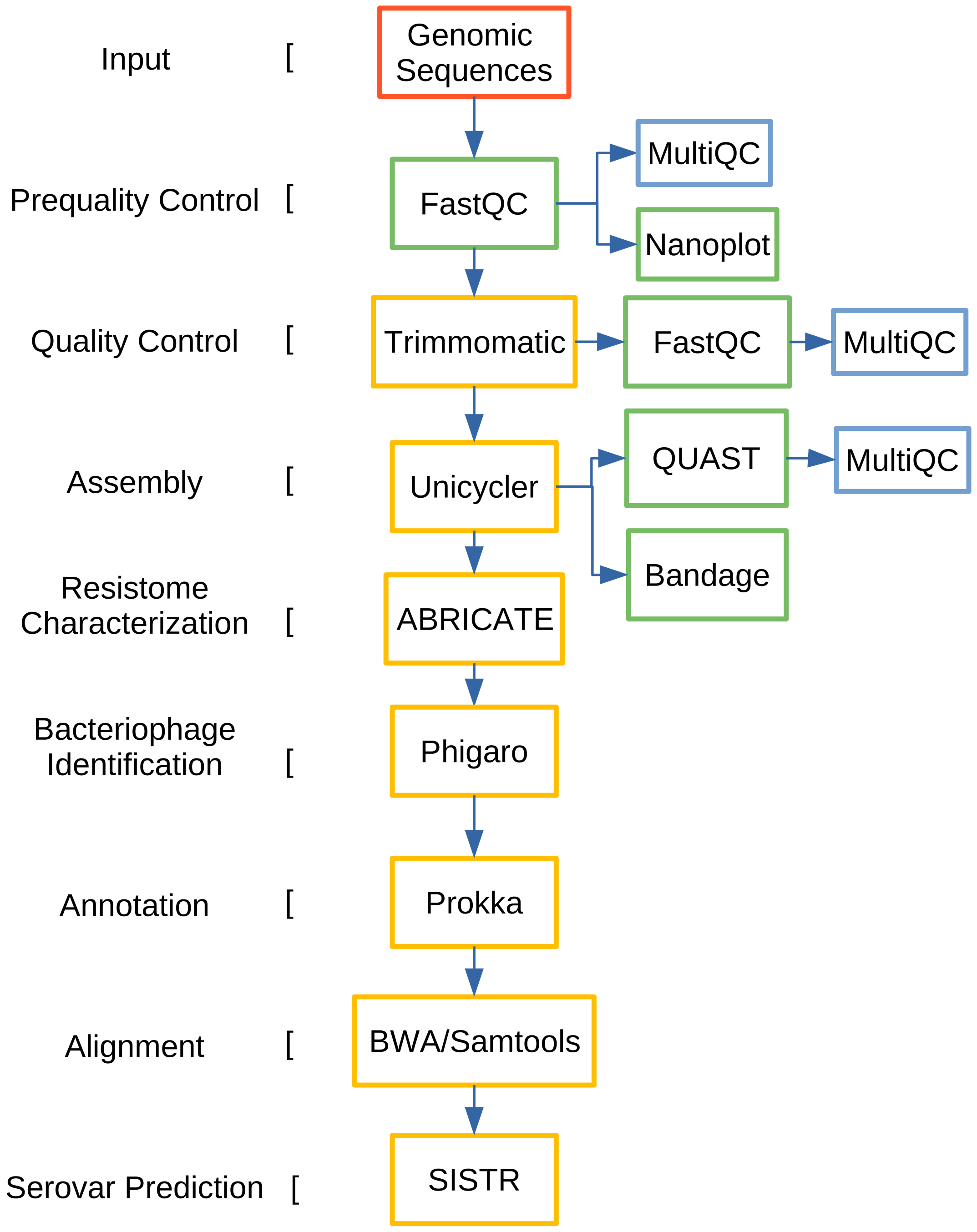

Figure S3. Key processes in Reads2Resistome pipeline.

4) Supplementary Tables

Table S2. Summary of isolates used for Reads2Resistome pipeline testing

Bacterial strains	Strain ID	SRA accession no.	Sequencing platform	Coverage*
Escherichia coli	EC-IC	SRR11808523	Illumina	56.95
		SRR11808522	MinION	35.35
		SRR11808521	PacBio	28.08
Salmonella Heidelberg	SH-IC	SRR11808520	Illumina	20.25
		SRR11808519	MinION	16.69
		SRR11808518	PacBio	33.14

* Coverage estimated from total quality-controlled bases divided by the genome size

(*E. coli*: 4800000bp , *S*. Heidelberg: 4600000bp)

Table S3. Summary of assembly resources and run-time under various assembly conditions

Isolates included in run	Assembly Method	Elapsed time	--threads (option)	CPU-hours
EC-IC Illumina; SH-IC Illumina	Short Read	16m 13s	64	0.6
EC-IC MinION; EC-IC PacBio; SH-IC MinION; SH-ICPacBio	Hybrid	4h 31m 45s	64	5.3
EC-IC MinION; EC-IC PacBio; SH-IC MinION; SH-IC PacBio	Long Read	23m 46s	64	0.9

Table S4. Summary of evaluation for assembled isolates under various assembly conditions

		Short Read	Hybrid		Long Read	
		Illumina	MinION	PacBio	MinION	PacBio
Isolate	Assembly Metrics and Annotated Features					
EC-IC	No. contigs	220	11	10	16	6
	Largest Contig (bp)	143515	3902334	3890483	4876904	3256237
	Total Length (bp)	5082305	5292549	5286973	5266949	5421221
	N50 (bp)	42032	3902334	3890483	4876904	3256237
	L50	36	1	1	1	1
	GC (%)	50.54	50.38	50.36	50.05	48.86
	tRNAs	80	90	90	32	12
	CRISPRs	1	1	1	0	0
	Predicted CDS	4765	5041	5004	4826	4837
	Annotated Genes	2277	2297	2295	430	185
SH-IC	No. contigs	57	19	8	90	5
	Largest Contig (bp)	460444	2046586	4750196	64272	2033197
	Total Length (bp)	4844513	4869998	4899506	1448734	5035134

	N50 (bp)	213070	1175028	4750196	16758	1502365
	L50	9	2	1	28	2
	GC (%)	52.1	52.08	52.1	50.44	50.52
	tRNAs	77	76	82	2	30
	CRISPRs	3	3	3	25	1
	Predicted CDS	4554	4573	4581	1387	5976
	Annotated Genes	2037	2044	2043	87	213

Table S5. Summary of resistome characterization for assembled isolates under various assembly conditions

		Short Read	Hybrid		Long Read	
		Illumina	MinION	PacBio	MinION	PacBio
Isolate	Database	Number of identified elements				
EC-IC	ARG-ANNOT	8	8	8	7	8
	CARD	48	48	48	44	43
	MEGARes	58	58	58	53	52
	NCBI	4	4	4	3	4
	PlasmidFinder	7	7	7	5	5
	ResFinder	4	4	4	3	4
	VirulenceFinder	70	72	72	55	55
SH-IC	ARG-ANNOT	8	8	8	6	5
	CARD	29	30	30	6	9
	MEGARes	36	37	37	6	16
	NCBI	4	4	4	4	3
	PlasmidFinder	3	3	3	2	2
	ResFinder	5	5	5	5	4
	VirulenceFinder	105	105	106	44	99

Table S6. Drug classes, identified through the ResFinder output of acquired AMR genes, which significantly differed in proportion between human clinical isolates and chicken production isolates as determined by the Wilcoxon rank-sum test. Chicken production isolate average proportions were compared against human clinical isolates. Genes conferring resistance to drug classes were enumerated for each isolate and a proportion was calculated using the total number of genes in the study population conferring resistance to a given drug class. P value adjustment performed by the Benjamini-Hochberg false discovery rate correction.

**Drug Class**	**Adjusted p value**
cephalosporin	1.99E-20
penam	6.33E-20
penem	5.85E-17
monobactam	5.85E-17
diaminopyrimidine antibiotic	3.83E-12
macrolide antibiotic	6.94E-09
disinfecting agents and intercalating dyes	5.70E-08
sulfonamide antibiotic	0.00033
carbapenem	0.0019
fluoroquinolone antibiotic	0.015
phenicol	0.042
aminoglycoside antibiotic	0.49
tetracycline antibiotic	0.85
cephamycin	1
streptogramin antibiotic	1
lincosamide antibiotic	1
disinfecting agents	1
fosfomycin	1
peptide antibiotic	1
benzalkonium chloride	1
rhodamine	1

Table S7. Drug classes, identified through RGI (CARD) output of AMR genes, which significantly differed in proportion between human clinical isolates and chicken production isolates as determined by the Wilcoxon rank-sum test. Chicken production isolate average proportions were compared against human clinical isolates. Genes conferring resistance to drug classes were enumerated for each isolate and a proportion was calculated using the total number of genes in the study population conferring resistance to a given drug class. P value adjustment performed by the Benjamini-Hochberg false discovery rate correction.

**Drug Class**	**Adjusted p value**
diaminopyrimidine	1.53E-42
fluoroquinolone	1.75E-41
macrolide	8.25E-32
cephamycin	1.53E-27
monobactam	1.49E-18
penem	9.27E-18
fosfomycin	6.42E-17
aminoglycoside	6.76E-11
sulfonamide	2.84E-09
elfamycin	1.10E-05
phenicol	0.00027
peptide	0.00029
nucleoside	0.0069
streptogramin	0.015
rifamycin	0.21
lincosamide	0.24
tetracycline	0.39
aminocoumarin	0.44

cephalosporin		1
glycylcycline		1
penam		1
triclosan		1
glycopeptide		1
benzalkoniumchloride		1
rhodamine		1
nitrofuran		1
carbapenem		1
acridinedye		1
oxazolidinone		1
pleuromutilin		1
nitroimidazole	NaN	

Table S9. Confusion matrix resulting from Random Forests classification on filtered presence/absence table of identified AMR and virulence factors (Table S1). All 791 chicken production and human clinical isolates were reclassified into their respective clusters which were identified through hierarchical clustering. Rows indicate reclassified isolates and columns original hierarchical cluster.

**Hierarchical Cluster**	**A**	**B**	**Error**
**A**	**644**	**0**	**0**
**B**	**0**	**130**	**0**

Table S10. Confusion matrix resulting from Random Forests classification on filtered presence/absence table of identified AMR and virulence factors (Table S1). All 791 chicken production and human clinical isolates were reclassified into their respective host sources. Rows indicate reclassified isolates, columns original host, and red numbers indicate misclassified.

Host	Chicken Production	Human Clinical	Error
**Chicken Production**	**447**	**5**	**0.011**
**Human Clinical**	**19**	**323**	**0.0556**

Table S11. Random Forests classification misclassified host results. All 791 chicken production and human clinical isolates were reclassified into their respective Host sources. Random Forests classification was performed on the filtered presence/absence table of identified AMR and virulence factors (Table S1).

Random Forests Host Predictions

**Isolate**	**Predicted Host**	**Actual Host**	**Isolation source**	**Phylogroup**	**Hierarchical Cluster**
SRR10687714	Human	Chicken Pro.	Chicken Thighs	F	A
SRR10687982	Human	Chicken Pro.	Chicken Wings	A	A
SRR8590847	Human	Chicken Pro.	Chicken	B2	A
SRR9984631	Human	Chicken Pro.	Chicken Wings	B1	A
SRR9984720	Human	Chicken Pro.	Chicken Wings	B1	A
SAMN09981238	Chicken Pro.	Human	Feces	A	A
SAMN10620159	Chicken Pro.	Human	Sepsis	A	A
SAMN10722958	Chicken Pro.	Human	Rectal	A	A
SAMN10722963	Chicken Pro.	Human	Rectal	A	A
SAMN10722966	Chicken Pro.	Human	Rectal	A	A
SAMN09981239	Chicken Pro.	Human	Feces	B1	A
SAMN09981247	Chicken Pro.	Human	Feces	B1	A
SAMN09981249	Chicken Pro.	Human	Feces	B1	A
SAMN09981263	Chicken Pro.	Human	Feces	B1	A
SAMN09981265	Chicken Pro.	Human	Feces	B1	A
SRR10728218	Chicken Pro.	Human	Blood	B1	A
SAMN09981305	Chicken Pro.	Human	Feces	B1	A
SAMN09981307	Chicken Pro.	Human	Feces	B1	A
SAMN09981302	Chicken Pro.	Human	Feces	B2	A
SAMN10722961	Chicken Pro.	Human	Rectal	B2	A

SAMN10620143	Chicken Pro.	Human	Sepsis	D	A
SAMN09981237	Chicken Pro.	Human	Feces	D	A
SRR6892704	Chicken Pro.	Human	Feces	D	A
SRR6892705	Chicken Pro.	Human	Feces	D	A

Table S12. RGI-identified antimicrobial resistance genes with mutations conferring resistance

Gene w/mutation	Mutation
Escherichia coli EF-Tu mutants conferring resistance to Pulvomycin	R234F
Escherichia coli parC conferring resistance to fluoroquinolones	S80I
Escherichia coli UhpT with mutation conferring resistance to fosfomycin	E350Q
Escherichia coli PtsI with mutation conferring resistance to fosfomycin	V25 I
Escherichia coli gyrA conferring resistance to fluoroquinolones	S83L (30 isolates)<br>S84L (2 isolates)<br>D87Y (275 isolates)
Escherichia coli GlpT with mutation conferring resistance to fosfomycin	E448K
Haemophilus influenzae PBP3 conferring resistance to beta-lactam antibiotics	D350N
Escherichia coli gyrA conferring resistance to triclosan	D87G
Escherichia coli cyaA with mutation conferring resistance to fosfomycin	S352T
Escherichia coli marR mutant conferring antibiotic resistance (fluoroquinolone antibiotic; cephalosporin; glycylcycline; penam; tetracycline antibiotic; rifamycin antibiotic; phenicol antibiotic; triclosan)	Y137H
Escherichia coli soxR with mutation conferring antibiotic resistance (fluoroquinolone antibiotic; cephalosporin; glycylcycline; penam; tetracycline antibiotic; rifamycin antibiotic; phenicol antibiotic; triclosan)	R20H and G121D
Escherichia coli soxS with mutation conferring antibiotic resistance (fluoroquinolone antibiotic; monobactam; carbapenem; cephalosporin; glycylcycline; cephamycin; penam; tetracycline antibiotic; rifamycin antibiotic; phenicol antibiotic; triclosan; penem)	n/a
Escherichia coli acrR with mutation conferring multidrug antibiotic resistance (fluoroquinolone antibiotic; cephalosporin; glycylcycline; penam; tetracycline antibiotic; rifamycin antibiotic; phenicol antibiotic; triclosan)	n/a

Table S13. Confusion matrix resulting from Random Forests classification on filtered presence/absence table of identified AMR and virulence factors (Table S1). All 791 chicken production and human clinical isolates were reclassified into their respective ClermonTyper identified phylogroups sources. Rows indicate reclassified isolates, columns original host, and red numbers indicate misclassified.

Phylogroup	A	B1	B2	C	clade I	D	E	E or clade I	F	G	Unknown	Error
A	117	14	0	0	0	1	0	0	0	0	0	0.11
B1	5	177	0	1	0	0	0	0	0	0	0	0.03
B2	0	0	184	0	0	0	0	0	0	0	0	0
C	0	11	0	12	0	0	0	0	0	0	0	0.48
clade I	0	0	0	0	8	0	0	0	0	0	0	0
D	0	0	0	0	0	103	0	0	0	0	0	0
E	0	0	0	0	0	4	71	0	0	0	0	0.05
E or clade I	0	0	0	0	0	3	1	0	0	0	0	1
F	0	0	0	0	0	0	0	0	25	1	0	0.04
G	0	0	0	0	0	1	0	0	0	54	0	0.02
Unknown	1	0	0	0	0	0	0	0	0	0	0	1

Table S14. Virulence factor-associated functions which significantly differed in proportion between human clinical isolates and chicken production isolates as determined by the Wilcoxon rank-sum test. Virulence genes associated with each function were enumerated for each isolate and a proportion was calculated using the total number of genes in the study population with the given function. P value adjustment performed by the Benjamini-Hochberg false discovery rate correction.

**VFDB Fcn**	**Adjusted p value**
Autotransporter	7.95E-42
Toxin	2.54E-28
Adherence	1.08E-23
Invasion	6.34E-23
LEE-encoded-TTSS-effectors	1.79E-12
Iron.uptake	5.33E-11
Non-LEE-encoded-TTSS-effectors	6.53E-09
Secretion system	0.00029
Protease	0.051
Immune evasion	0.067
Fimbrial adherence determinants	0.078

Table S15. Proportion (%) of virulence factor-associated functions across identified phylogroups. The set of functions for each gene was counted and summed for all isolates in a given phylogroup. Virulence genes associated with each function were enumerated for each isolate and a proportion was calculated using the total number of genes in the study population with the given function.

Phlyogroup	Adherence	Protease	Autotransporter	Invasion	Iron uptake	LEE-encoded TTSS effectors	Non-LEE encoded TTSS effectors	Secretion system	Toxin	Immune evasion	Fimbrial adherence determinants
**A**	13.41	1.22	0.04	11.45	22.31	8.72	10.31	6.47	1.59	5.60	55.94
**B1**	14.93	3.06	0.11	0.36	24.15	6.32	10.22	7.34	3.57	2.37	57.14
**B2**	19.69	0.55	4.40	35.59	45.98	0.86	0.19	5.42	5.75	2.20	56.99
**C**	16.73	0.00	0.00	0.00	33.75	0.00	7.00	4.93	0.18	0.00	57.14
**clade I**	16.63	0.00	0.00	36.11	32.50	0.00	15.51	6.32	3.13	0.00	57.14
**D**	16.82	0.00	0.83	24.49	38.57	6.66	17.76	6.76	2.59	3.56	54.51
**E**	17.81	16.00	3.93	12.30	27.75	67.05	47.06	14.58	24.22	0.89	56.95
**E or clade I**	15.63	0.00	0.00	11.11	31.25	32.14	20.37	15.99	4.17	0.00	53.57
**F**	16.83	0.77	0.77	33.76	42.16	0.00	11.61	6.13	1.60	0.00	57.14
**G**	16.83	0.77	0.77	33.76	42.16	0.00	11.61	6.13	1.60	0.00	57.14
**Unknown**	7.14	0.00	0.00	11.11	36.25	0.00	12.96	6.40	4.17	0.00	57.14

5) Supplemental References

[1] P. DI Tommaso, M. Chatzou, E. W. Floden, P. P. Barja, E. Palumbo, and C. Notredame, “Nextflow enables reproducible computational workflows,” Nature Biotechnology, vol. 35, no. 4. Nature Publishing Group, pp. 316–319, Apr. 11, 2017, doi: 10.1038/nbt.3820.

[2] G. M. Kurtzer, V. Sochat, and M. W. Bauer, “Singularity: Scientific containers for mobility of compute,” PLoS One, vol. 12, no. 5, p. e0177459, May 2017, doi: 10.1371/journal.pone.0177459.

[3] A. M. Bolger, M. Lohse, and B. Usadel, “Trimmomatic: A flexible trimmer for Illumina sequence data,” Bioinformatics, vol. 30, no. 15, pp. 2114–2120, 2014, doi: 10.1093/bioinformatics/btu170.

[4] W. De Coster, S. D’Hert, D. T. Schultz, M. Cruts, and C. Van Broeckhoven, “NanoPack: Visualizing and processing long-read sequencing data,” Bioinformatics, vol. 34, no. 15, pp. 2666–2669, 2018, doi: 10.1093/bioinformatics/bty149.

[5] R. R. Wick, L. M. Judd, C. L. Gorrie, and K. E. Holt, “Unicycler: Resolving bacterial genome assemblies from short and long sequencing reads,” PLoS Comput. Biol., vol. 13, no. 6, 2017, doi: 10.1371/journal.pcbi.1005595.

[6] A. Bankevich et al., “SPAdes: A new genome assembly algorithm and its applications to single-cell sequencing,” J. Comput. Biol., vol. 19, no. 5, pp. 455–477, 2012, doi: 10.1089/cmb.2012.0021.

[7] H. Li, “Minimap and miniasm: Fast mapping and de novo assembly for noisy long sequences,” Bioinformatics, vol. 32, no. 14, pp. 2103–2110, 2016, doi: 10.1093/bioinformatics/btw152.

[8] R. Vaser, I. Sović, N. Nagarajan, and M. Šikić, “Fast and accurate de novo genome assembly from long uncorrected reads,” Genome Res., vol. 27, no. 5, pp. 737–746, May 2017, doi: 10.1101/gr.214270.116.

[9] T. Seemann, “Prokka: Rapid prokaryotic genome annotation,” Bioinformatics, vol. 30, no. 14, pp. 2068–2069, Jul. 2014, doi: 10.1093/bioinformatics/btu153.

[10] T. Seemann, “ABRICATE,” Github, [Online]. Available: https://github.com/tseemann/abricate.

[11] P. Ewels, M. Magnusson, S. Lundin, and M. Käller, “MultiQC: Summarize analysis results for multiple tools and samples in a single report,” Bioinformatics, vol. 32, no. 19, pp. 3047–3048, 2016, doi: 10.1093/bioinformatics/btw354.

[12] C. E. Yoshida et al., “The salmonella in silico typing resource (SISTR): An open web-accessible tool for rapidly typing and subtyping draft salmonella genome assemblies,” PLoS One, vol. 11, no. 1, 2016, doi: 10.1371/journal.pone.0147101.

[13] M. Hunt, C. Newbold, M. Berriman, and T. D. Otto, “A comprehensive evaluation of assembly scaffolding tools,” Genome Biol., vol. 15, no. 3, p. R42, Mar. 2014, doi: 10.1186/gb-2014-15-3-r42.

[14] R. R. Wick, M. B. Schultz, J. Zobel, and K. E. Holt, “Bandage: Interactive visualization of de novo genome assemblies,” Bioinformatics, vol. 31, no. 20, pp. 3350–3352, 2015, doi: 10.1093/bioinformatics/btv383.

[15] E. V. Starikova et al., “Phigaro: High-throughput prophage sequence annotation,” Bioinformatics, vol. 36, no. 12, pp. 3882–3884, 2020, doi: 10.1093/bioinformatics/btaa250.
